# C-C Chemokine Receptor 7 in Cancer

**DOI:** 10.3390/cells11040656

**Published:** 2022-02-14

**Authors:** Colin A. Bill, Christopher M. Allen, Charlotte M. Vines

**Affiliations:** Department of Biological Sciences, The University of Texas at El Paso, El Paso, TX 79968, USA; cabill@utep.edu (C.A.B.); cmallen2@miners.utep.edu (C.M.A.)

**Keywords:** cancer, survival, metastases, C-C chemokine receptor 7

## Abstract

C-C chemokine receptor 7 (CCR7) was one of the first two chemokine receptors that were found to be upregulated in breast cancers. Chemokine receptors promote chemotaxis of cells and tissue organization. Since under homeostatic conditions, CCR7 promotes migration of immune cells to lymph nodes, questions immediately arose regarding the ability of CCR7 to direct migration of cancer cells to lymph nodes. The literature since 2000 was examined to determine to what extent the expression of CCR7 in malignant tumors promoted migration to the lymph nodes. The data indicated that in different cancers, CCR7 plays distinct roles in directing cells to lymph nodes, the skin or to the central nervous system. In certain tumors, it may even serve a protective role. Future studies should focus on defining mechanisms that differentially regulate the unfavorable or beneficial role that CCR7 plays in cancer pathophysiology, to be able to improve outcomes in patients who harbor CCR7-positive cancers.

## 1. Introduction

**Figure 1:**
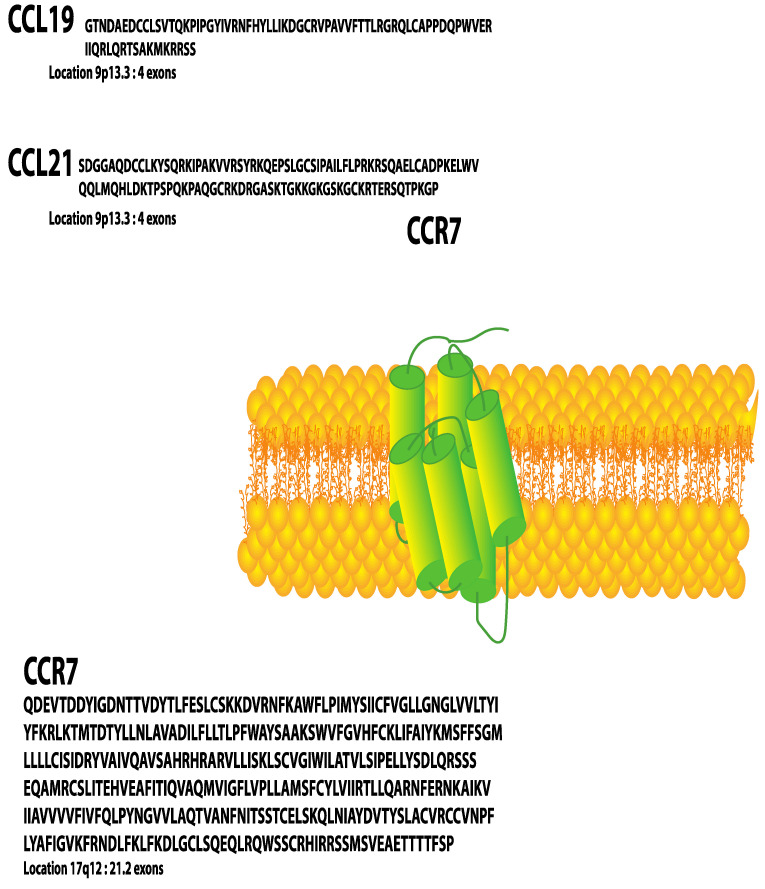
CCR7 (Uniprot Available (https://www.uniprot.org/uniprot/P32248) P32248 (accessed on 2 February 2022) [amino acids 25–378]) and its ligands CCL19 (Uniprot Available (https://www.uniprot.org/uniprot/Q99731) Q99731 (accessed on 2 February 2022) [amino acids 22–98]) and CCL21 (Uniprot. Available (https://www.uniprot.org/uniprot/O00585) O00585 (accessed on 2 February 2022) [amino acids 24–134]).

C-C chemokine receptor 7 (CCR7) was the first lymphocyte-specific G protein-coupled receptor (GPCR) identified and was originally named Epstein–Barr virus (EBV)-induced gene 1 (EBI1) since it was upregulated in EBV-infected Burkitt’s lymphoma B cells [1]. Later, CCR7 was re-identified in a screen for chemokine receptors of EBV-infected cells and at that point named Burkitt’s lymphoma receptor 2 (BLR2) [2]. Two ligands for CCR7 have been identified, CCL19 (MIP3β/ELC/CKβ11/EBI1-Ligand/SCYA19/exodus 3) [3,4,5,6,7,8,9] and CCL21(SLC/6ckine/SCYA21/exodus 2) [7,9,10,11,12] (Figure 1). These small polypeptides, 8 and 13 kDa, respectively, promote migration of CCR7-expressing activated dendritic cells and naïve T cells [3] to and within secondary lymphoid organs. Later studies revealed that CCR7 and its ligands could also be upregulated to promote trafficking of activated B cells [13], macrophage progenitors [14], NK cells [12] and central memory T cells to secondary lymphoid organs and during thymopoiesis of thymocytes within the thymus [15,16]. Since many chemokines and their cognate GPCRs have been described and named by multiple laboratories, to eliminate confusion, a Keystone Conference was convened which re-named chemokines and their receptors based on the structure of their ligands [17].

**Figure 2:**
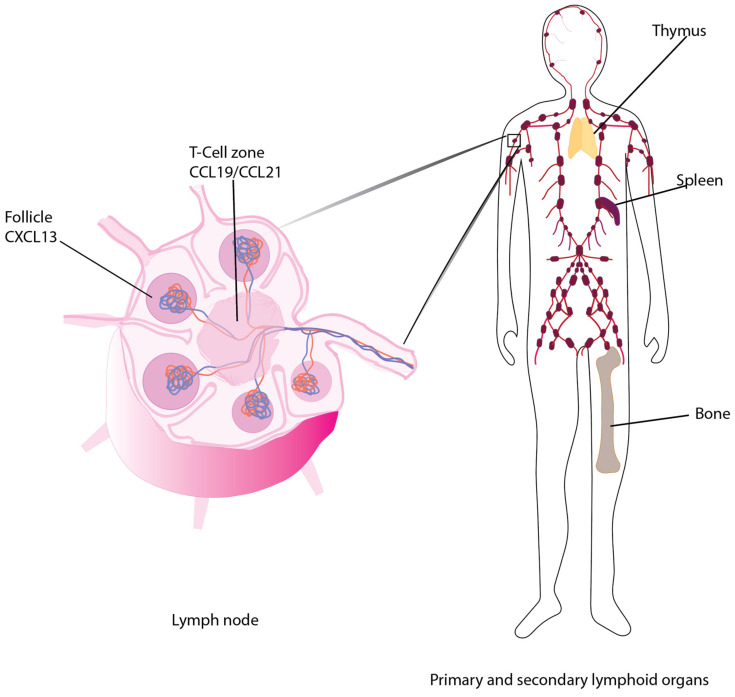
CCR7 promotes chemotaxis of cells to the T-cell zones of secondary lymphoid organs.

CCL19 and CCL21 ligands are constitutively expressed by stromal cells within primary and secondary lymphoid organs and are, therefore, described as homeostatic chemokines [18]. CCL21 is also expressed on the surface of high endothelial venules of mice and lymphatic endothelium of mice and humans [19]. The gene encoding human CCR7 is localized to human chromosome 17q12-21.2 and is composed of three exons, which encode 378 amino acids [20] (Figure 2). The mouse homolog is encoded on chromosome 11 and encodes a protein that shares 86% identity with human CCR7. Both human and mouse CCR7 induce chemotaxis to CCL19 and CCL21. In addition, there are two CCL21 homologs in mice—CCL21-Ser/CCL21a and CCL21-Leu/CCL21b. The high levels of homology of mouse and human receptors and ligands make mouse models useful for studying CCR7 function relevant to cancer in humans. Although this review will not discuss atypical chemokine receptors (ACKRs), it is important to note that ACKR4 can act as a scavenger receptor, binding and internalizing both CCL19 and CCL21, thus reducing their availability to bind and activate CCR7 (Gosling, 2000).

Under homeostatic conditions, CCR7-CCL19 and CCL21 contribute to the organization of secondary lymphoid structures via regulating recruitment of immune cells to the T-cell zones within the lymph nodes and spleen [19,21] and the activated B cells, macrophages and dendritic cells to the T-cell/follicle border [6,21,22,23,24]. Mice lacking CCR7, CCL19 or CCL21 expression due to homozygous deletion (CCR7^−/−^ or CCL19^−/−^ mice) [13,22] or the paucity of lymph node (plt/plt) mouse, in which spontaneous mutations led to the loss of a functional CCL19 and CCL21-ser genes, although the second CCL21 gene, CCL21-leu is functional [25], provide tools for studying the roles of each ligand or receptor in the metastasis of cancer or during an immune response. 

In cancer, chemokines in general can play multiple roles within the network of inflammatory mediators which include promoting infiltration of tumors by immune cells, lymphangiogenesis and angiogenesis [26,27]. CCR7, however, plays a unique role in tumorigenesis by targeting tumor cell metastasis to the T-cell zones of lymph nodes [28,29] (Figure 2). CCL19 and CCL21 are differentially distributed to distinct locations in host tissues, primarily due to the extended C-terminus of CCL21, which contains eight positively charged lysines. These amino acids are bound by glycosaminoglycans via the heparin-binding domain to form a gradient that orchestrates lymphocyte or tumor cell recruitment to secondary lymphoid organs [30]. Since the presence of lymph node metastasis can worsen the prognosis of a malignancy, it could be concerning that certain cancer cells upregulate their CCR7 and detach from the primary tumor, perhaps in response to the CCL21 gradient, which promotes directed migration (chemotaxis) to and through lymphatic vessels. Moreover, metastatic cancer cells can express CCR7 ligands that, in the presence of interstitial flow, create autologous, transcellular chemokine gradients that induce cancer cell chemotaxis to draining lymphatics [31].

Our understanding of how tumors use chemokines to metastasize to different tissues gained momentum in 2000, when Dr. Anja Müeller and Dr. Albert Zlotnik reported that two chemokine receptors and their ligands could promote chemotaxis of tumor cells [32]. Antagonizing these receptors and/or ligands provided novel platforms for cancer therapeutics: CCR7/CCL21 for lymph node metastases, and CXCR4/CXCL12 for lung, liver, bone marrow and brain metastases. However, anti-CCR7 therapy is not necessarily advantageous; for example, a study adapted a virus middle T-antigen (PyV MT) syngeneic adenocarcinoma mouse model to examine the effects of CCR7 expression on mammary tumor cell metastasis [33]. They found that similar to results that had been reported previously in humans, the presence of CCR7 in the tumors provided a significant improvement (*p* = 0.00027) in disease-free survival when compared to women with no/low CCR7 expression in their tumors [34]. 

The roles of CCR7 in cancers are complex, and the reports are inconsistent. At times, this can be related back to differences in the scientific approaches that were used or lack of appropriate controls, and we try to highlight these differences. In addition, in our review, we discuss the reported clinical and animal study data and provide a summary of areas that are promising for further investigation. 

## 2. Breast Cancers

After skin cancer, breast cancer is the most common female malignancy in the United States, with approximately 1 in 8 women developing the disease over their lifetimes [35]. In this section, expression of CCR7 during cancer initiation, progression, metastasis and at diagnosis will be discussed. Initial studies reported that CCR7 mRNA levels were elevated in seven human breast cancer cell lines when compared to normal primary mammary epithelial isolates [32]. In addition, CCR7 mRNA levels were elevated in 12 primary human invasive lobular or ductal breast carcinomas when compared to normal mammary gland tissues [32]. CCL21 stimulation of MDA-MB-231 and MDA-MB-361 human breast cancer cell lines in vitro enhanced intracellular filamentous actin, while inducing pseudopodia and invasive responses. From this result, it could be inferred that the CCR7/CCL21 signaling may promote breast cancer chemotaxis to CCL21-producing organs such as lymph nodes, although in this small study, CCR7 mRNA levels did not correlate with breast cancer TNM staging for tumor size (T), spread to lymph nodes (N), and presence of metastasis (M); nor was expression of CCR7 protein on the cell surface confirmed [32].

During the development of breast cancer, there exists a potential link between endothelins and CCR7 [36]. Since endothelin-1 is one of the body’s most potent vasoconstrictors, it may regulate the perfusion of the tumor with leukocytes, which are found in the blood. Endothelin-1 is one member of a family of three 21 amino acid peptides, two of which activate either the (endothelin receptor A) ET_A_ or ET_B_ GPCRs [37,38]. Expression of endothelins and their receptors is higher in malignant breast tissue compared to non-cancerous breast tissues [36,38]. In vitro, endothelin activation of ET_A_ correlated with increased CCR7 cell surface expression in MCF-7, SKBR3 and MDA-MB-231 human breast cancer cell lines, which enhanced MCF-7 invasion of Matrigel towards CCL21 or CCL19 only in the presence of endothelin. When an anti-CCR7 function-blocking antibody was used in this invasion assay, it reduced invasion from 10 to 5 cells/high powered field. Unfortunately, since the data was extremely variable, the significance of this observation was unclear [36]. When levels of CCR7 mRNA were measured in primary human invasive breast cancers, patients with lymph node metastases showed elevated ET-1 and CCR7 expression. This could be due to the presence of CCR7-expressing immune cells in the tumor, cells which normally express ET-1, in the presence of the ET-1-mediated vasoconstriction. It was postulated that endothelin mediated stabilization of hypoxia-inducible factor 1 (HIF-1), which increased CCR7 expression. In the future, it would be interesting to examine the levels of CCR7 in the actual tumor cells of breast cancer patients in the absence of infiltrating immune cells to better confirm that ET-1 combined with CCR7 promotes migration of tumor cells to the lymph nodes. Alternatively, ET-1-induced vasoconstriction could trap CCR7-expressing dendritic cells or macrophages that traffic the tumor cells to the lymph nodes via the vasculature. 

In addition to CCR7, macrophages can express different pro-inflammatory eicosanoids such as prostaglandin E2 (PGE2). In turn, PEG2 promotes surface expression of CCR7 and subsequent ligand-dependent migration of dendritic cells [39] via the prostaglandin E2 receptors (EP2 and EP4) [40,41]. Cyclooxygenase-2 (COX-2), a member of the cyclooxygenase enzyme family, mediates the synthesis of prostaglandins. Overexpression of COX-2 is commonly reported in many types of cancer, including breast cancers, where it is typically associated with a poor prognosis [42,43,44,45,46,47,48]. In breast cancer, CCR7 expression was significantly associated with COX-2 expression (*p* = 0.008) [48]. In these studies, ectopic expression of COX-2 in MCF-7 breast cancer cells resulted in upregulation of CCR7 on the cell surface, while knockdown of COX-2 by small hairpin RNA led to reduced CCR7 expression [48]. Therefore, it is not surprising that COX-2 expression correlates with lymph node metastasis of breast cancer [44,48]. Subsequently, it was shown that COX-2 and its metabolite PGE2 promote CCR7 expression via AKT-mediated phosphorylation of the Sp1 transcription factor, which can then bind the CCR7 promoter. The expression of COX-2, CCR7, and the prostaglandin E2 receptors (EP2 and EP4) correlated with lymph node metastasis of breast cancer [47]. Many COX-2 inhibitors have been investigated for their anti-tumor effects, and may reduce the numbers of CCR7-directed metastases to the lymph nodes [49]. 

In addition to SP-1, other transcription factors such as Ets-1, have been shown to promote CCR7 expression in (triple negative) basal cell breast tumors. In these studies, Ets-1 had two roles. First, this transcription factor was found to play an important role in regulating CCR7 expression in T helper cells [50], since CCR7 expression was reduced in Ets-1-deficient T cells following CD3/CD28 stimulation of the T-cell receptor. Ets-1 was also shown to bind to the CCR7 promoter and there was a good correlation between Ets-1 expression and CCR7 expression in basal-type breast cancer cell lines, such as MDA-MD-231, suggesting that Ets-1 is a likely mediator of CCR7 effects including cancer cell migration [50].

At diagnosis, the correlation between CCR7 expression and lymph node metastasis appears to be complex in breast cancer. While some studies report that CCR7 was a useful biomarker to predict lymph node metastasis of breast cancer, others do not. To some extent, however, the behavior of the tumor depends upon the type of breast cancer. Low-grade luminal A tumors expressed lower levels of CCR7 than more metastatic breast cancers. However, when luminal A tumors expressed CCR7, these cells did not migrate to lymphatic vessels even in the presence of CCL21, which was thought to be due to the side effects of hormones, TNF-α and epidermal growth factor [51]. This study suggested that CCR7 expression by itself is not always a good marker for lymphatic metastasis. In contrast, more aggressive luminal B breast cancers had high CCR7 expression levels, which correlated well with lymph node metastasis [52]. This correlation was even more evident in highly aggressive triple-negative breast cancers, where CCR7 was highly expressed in both cell lines and breast cancer tissue. Additionally, in a mouse model of murine 4T1 triple-negative breast cancer, when CCR7 was knocked down by shRNA, growth and invasive properties were curtailed, suggesting that CCR7 enhances metastasis via promoting tumor cell proliferation/invasion at the metastatic site [53]. Furthermore, when modified antibodies were used to block CCR7 function in the 4T1 mouse model, the concomitant reduction in CCR7 reduced triple-negative breast cancer lymphatic metastasis [54]. The most aggressive type of primary breast cancer, inflammatory breast cancer has a poor prognosis. In a study of inflammatory breast cancer, immunohistochemical analysis of receptor paraffin-embedded tumor tissue sections revealed that ~23% of inflammatory breast cancer samples were positive for CCR7, which correlated with a decreased 5 year overall survival for CCR7-positive patients (20%) versus 41.9% for CCR7-negative patients [55]. The expression level of CCR7 in breast tumors can be low and in one study, CCR7 was present in only 10% of patients [56]. CCR7 metastasis may preferentially home to skin and bone. In one study, while 27% of bone metastases expressed CCR7, visceral sites lacked CCR7(+) metastasis, clearly indicating a preference of CCR7 for bone metastasis [56]. Similarly, in a separate study, although only 11% of skin metastases expressed CCR7 in primary breast cancer patients, in a 13 year follow-up of study, none of the CCR7-negative primary breast cancer patients had skin metastases, which was statistically significant [57]. In a contrasting study, when human breast cancer immunohistology specimens were examined, no correlation was found between CCR7 cytoplasmic staining and lymph node positivity [58]. Unfortunately, in this study, which relied heavily on an anti-CCR7 antibody, the validation of this CCR7 antibody relied on a Western blot, which lacked a negative control, making the data impossible to interpret, since it was impossible to confirm that the antibody was specific for CCR7. Overall, the cells that express CCR7 within breast cancer tissue are often not clearly defined; however, immunohistochemistry of paraffin-embedded tissue sections suggested that CCR7 can be expressed by spindle-shaped stromal cells in different types of breast cancer [59]. In this study, expression of CCR7 was not associated with a significant change in overall patient survival [59]. Taken together, while these studies demonstrate that CCR7 seems to reliably predict the presence of lymph node metastases in more aggressive breast tumors, it is unclear whether CCR7 can be linked to patient survival in all breast cancers. In the future, it will be important to correlate stage of progression with the types of cells within a tumor that express CCR7.

To identify other factors that may predict lymph node metastasis, microarray analysis of primary breast cancer has been used. In these studies corresponding lymph nodes similar to CCR7, EGFR was highly expressed in tumors which metastasized to lymph nodes [60]. In addition, EGFR ligands were expressed at elevated levels in metastatic breast tumors compared to primary tumors. Kaplan–Meier survival plots indicated that CCR7- and EGFR-expressing breast tumors were associated with a shorter survival time compared to patients expressing low levels of the receptors [60]. A similar analysis of triple-negative breast cancer tissue samples reported that when CCR7 expression was mainly found in the cytoplasm, there was a significant elevation in local tumor recurrence compared to tumor that did not show such CCR7 localization [61]. Analysis of patient survival reported that despite the higher local recurrence level, there was no difference in 5 year survival rates for triple-negative breast cancer patients unlike what had been observed for all types of invasive ductal breast cancers [60,61]. These data could indicate that the co-expression of CCR7 and EGFR have offsetting affects, where the trafficking of breast tumor cells to the lymph nodes allows for immune exposure, perhaps providing an opportunity for immune surveillance, deep within the T-cell zone of the lymph nodes. It will be important to examine the anti-breast cancer immune responses in women with CCR7(+) metastases within the lymph nodes. 

In addition to EGF, other growth factors and hormones have been studied as targets for treatment in the progression of breast cancers for over 30 years [62]. For example, HER2/neu (receptor tyrosine-protein kinase erbB-2) gene amplification is observed in approximately 15% of breast cancers. HER2/neu (Chr17q12-21) [62] and CCR7 (Figure 1) are located close together on chromosome 17 and CCR7 is co-amplified with HER2/neu in approximately 20% of cases [63], although approximately 4% of HER2 amplified breast cancer samples were associated with CCR7 genomic deletion [63]. In human paraffin-embedded tissue samples, there was a generally high correlation between lymph node-positive tumors and high CCR7 cytoplasmic staining [64,65]. This could be expected since, in immune cells, while CCR7 is a membrane receptor and upon ligand stimulation CCR7 undergoes endocytosis, it can be processed through the trans-Golgi network [66,67] and thus cytoplasmic staining is anticipated. In these studies, the correlation between lymph node positivity of breast cancers and relevant biomarkers was improved by including additional CXCR4 and HER2-neu, in the analysis, if they were present [65]. 

Tumor lymphangiogenesis is a key process in the lymphatic metastasis of tumors. Expression of VEGF-C and CCR7 were reported in human breast cancer tissue, leading to the promotion of lymphatic invasion [68]. Mechanistically, VEGF-C elevated CCL21 lymphatic secretion, resulting in the chemotactic migration of CCR7-expressing breast tumor cells towards lymphatic vessels, promoting proliferation, migration [69] and tube formation of primary lymphatic endothelial cells [68]. When VEGF-C was intradermally injected into C57/Bl6 mice, there was an upregulation of lymphatic CCL21. Furthermore, VEGF-C increased tumor cell invasion to lymphatic endothelial cells that could be prevented by blocking either CCL21 or CCR7 [69]. Thus, VEGF-C may render a more lymphatic invasive tumor cell phenotype via activation of the CCL21/CCR7 signaling axis. 

An important consideration in metastatic spread is the survival of cancer cells that have detached from the primary tumor. By inhibiting anoikis (programmed cell death due to cell detachment from neighboring cells or extracellular matrix), CCR7 can increase metastatic potential [70]. Using the highly invasive triple-negative breast cancer MDA-231 cell line, it was shown that CCR7 deregulated apoptosis without any ECM interactions both in vitro and in vivo, resulting in increased cell survival. Notably, CCR7-reduced anoikis occurred in highly aggressive breast cancer cells, but not in untransformed or non-metastatic cells [70]. In a related pathway, it was reported that sialyltransferases were overexpressed in human breast cancer tissue and cell lines that were associated with activation of extracellular signaling kinase (ERK) and AKT signaling and prevention of anoikis [70]. More importantly, CCL19/CCR7-induced breast cancer cell growth was found to be significantly repressed and anoikis increased when cells were treated with sialyltransferase inhibitors [71]. Transforming growth factor β (TGF-β)-activated protein kinase 1 (TAK1) is a protein that regulates cell viability, inflammation, and programmed necrosis (necroptosis) [72,73]. TAK1 expression is commonly elevated in breast cancer tissue and often linked to elevated levels of CCR7 expression. Activation of TAK1 was shown to increase expression of CCR7 and enhance lymph node invasion of triple-negative breast cancer cells [72]. Inhibition of the TAK1 binding protein, TAB1, reduced CCR7 expression and tumor size in animal studies with associated suppression of lymph node invasion and metastasis [72].

The epithelial to mesenchymal transition (EMT) is a well-established process important for cancer progression and metastasis [74]. High expression of CCR7 and the EMT markers, Slug and N-cadherin was reported for 60, 65 and 77% of tumors from primary breast cancer tissues obtained from sixty patients after radical mastectomy, which correlated with lymph node metastasis and breast cancer stage [75]. In vitro studies on breast cancer cell lines revealed that CCL21 stimulation enhanced cell invasive properties, an EMT phenotype, upregulated Slug and N-cadherin with concomitant reduction in E-cadherin. Conversely, CCR7 inhibition reversed the breast cancer cell migratory and EMT functions [75,76]. Furthermore, TGF-β1-induced EMT targets breast cancer cells to migrate towards lymphatic vessels, as opposed to blood vessels when analyzed in vivo and 3D culture systems [77]. This TGF-β1-mediated lymphatic migration was associated with CCL21 release from lymphatic endothelial cells and chemotaxis of CCR7-expressing breast cancer cells [77]. This process was mediated by the TGF-β1 signal transducer, Smad, although Smad-independent pathways acting via Ras or Wnt were identified in BALB/c mice models [77]. These reports were the first to indicate that CCR7 may be involved in reverting the phenotype of certain more aggressive breast cancers from epithelioid to a more mesenchymal behavior.

CCR7 expression can alter the metastatic destination of breast cancer cells. Using the mouse MMTV-PyVMT model (CCR7 negative) that had been selected for metastasis to the lungs, it was confirmed that after implantation of these mammary cancer cells into the mammary fat pad, all mice tested showed lung metastasis with no spread to the lymph nodes. In contrast, when MMTV-PyVMT cells were transiently transfected with a CCR7-expressing vector, metastasis to the lungs decreased (4/10 mice), whereas lymph node metastasis was found in 6/10 mice, indicating that CCR7 expression promoted lymph node metastasis [33]. It was further shown by in vitro studies using mammary cell lines that β1-integrin heterodimeric adhesion molecules mediated CCR7 migration after incubation with CCL19 or CCL21. Furthermore, CCR7-expressing tumor cells grew more rapidly than CCR7-negative tumor cells both in vivo within mammary fat pads and in three-dimensional in vitro culture systems [33]. Using a similar MMTV-PyMT-driven mouse model, a second study reported that CCR7 deletion delayed mammary tumor formation, likely via the loss of stem-like cells [78]. In a third, follow-up study, the group surmised that CCR7 activation turned on the Notch1 signaling pathway with concomitant elevation of the cancer stem cell population and thus loss of CCR7 produced attenuated Notch1 responses, reduced stem cell numbers and slowed tumor formation and growth [79]. Paradoxically, these results contrast with Buonamici et al., who rather than concluding that CCR7 activated Notch1, showed that CCR7 is downstream of Notch1 in T-cell acute lymphoblastic leukemia (see below) [80].

It is unclear whether persistent expression of CCR7 is required for targeting metastasis to lymph nodes. MicroRNAs, short non-coding RNAs typically 19–25 nucleotides in length, can bind to the 3′untranslated region (3′UTR) of their target mRNAs to promote their degradation or inhibit their translation [81,82]. Lethal-7 (Let-7) is a key developmental microRNA first identified in the nematode, *Caenorhabditis elegans* and subsequently found to be conserved amongst animals. A family member, miR-let-7a, reduces breast cancer migration/invasion by downregulating CCR7 expression. CCR7, CCL21 and miR-let-7a were detected in both breast cancer cell lines and patient breast cancer tissue [83]. miR-let-7a was shown to target the 3′UTR of CCR7, leading to CCR7 protein reduction, which could be reversed by inhibition of miR-let-7a [83]. In the future, these types of microRNAs may provide platforms for regulating CCR7 expression during growth and metastasis. 

Mutations are the driving force of cancer genesis and progression. A study of single-nucleotide polymorphisms of several chemokines and receptors did not find a significant correlation between CCR7 mutations and breast cancer susceptibility [84]. Although not well studied, there is evidence that splice variants of CCR7 can significantly positively or negatively affect the progression of breast cancer and patient survival, at least for the basal-like breast cancer subtype [85].

In this section, expression of CCR7 during cancer initiation, progression, metastasis and at diagnosis is discussed (Table 1). Clearly, in breast cancer, signaling through CCR7 can have different outcomes dependent upon the state of the cancer. Upregulation of CCR7 is induced by a number of factors [50,72], where it can promote behaviors that facilitate metastasis such as activation of actin and invasion [32,36]. Additionally, in luminal B breast cancers, CCR7 expression correlates with Notch to promote tumor growth or stemness [33,52,53,78]. Overall, expression of CCR7 can promote metastasis to bone, skin or lymph nodes; the mechanisms regulating the migration of tumors to different sites are unclear [56,57,65]. In most forms of breast cancer, expression of CCR7 correlates with decreased 5 year survival and tumor recurrence [60,61,65]. In the future, it may be prudent to examine the effects of receptor antagonists in animal models as potential platforms for development of pharmaceuticals. 

## 3. CCR7 in Genitourinary Cancers

Bladder cancer is the fourth most common cancer in men, although less common in women [35]. An initial assessment of CCR7 expression in cystectomy sections by immunohistochemistry of 119 patients found that CCR7 was overexpressed in 24% of urothelial cancers of the bladder; however, CCR7 was not associated with an aggressive form of cancer [86]. In T24 human bladder carcinoma cells, CCL21 activation of CCR7 promoted cell proliferation and migration mediated by increased levels of matrix metalloproteinases 2 and 9 (MMP-2 and MMP-9) [87]. This activated CCR7 response reduced apoptosis by increasing the pro-survival Bcl-2 protein and decreasing pro-apoptotic Bax proteins [87]. In a follow-up study using clinical samples and T-24 bladder cancer cell lines, the same group reported that the microRNA, miR-199a-5p, which targets CCR7 mRNA for deactivation, was downregulated in bladder cancers. As expected, the authors observed increased expression of CCR7, which correlated with increased expression of MMP-9. Furthermore, they observed that miR-199a-5p downregulation correlates with TMN stage (*p* < 0.0001) tumor invasion (*p* < 0.001), and lymph node metastasis (*p* < 0.001). Specifically, human bladder cancer tissues, when paired with normal tissues, expressed 3.36-fold lower levels of miR-199a-5p in tumor tissues and 5.2-fold lower levels of miR-199a-5p in the bladder cancer cell lines, when paired with a normal epithelial cell line. Expectantly, in the same tissues, CCR7 levels were 6.6-fold higher in the tumors and 10.53-fold higher in the cell lines, when paired with normal tissues. Mechanistic studies confirmed that exogenous miR-199a-5p bound to the 3′UTR of CCR7 but had no effect on CCR7 mRNA levels, suggesting that this microRNA functions to inhibit translation [88]. Ribosome-binding protein 1 (RRBP1), an endoplasmic reticulum membrane protein required for ribosome binding and protein transportation is a marker of some solid cancers and is highly expressed in bladder cancer cell lines compared to transformed non-cancerous urethral cells [89]. High expression of RRBP1 reduced the overall survival of patients with bladder cancer, which might, at least in part, be due to its effects on CCR7. While not well defined, RRBP1 knockdown led to an elevation in CCR7 mRNA as well; however, the levels of CCR7 protein decreased presumably due to reduced CCR7 mRNA translation in the low RRBP1 environment with the consequence of attenuated bladder cancer cell migration and invasion. Unfortunately, an experiment to add back RRBP1 was not conducted to validate the role of CCR7 in bladder cancer migration/invasion [89].

A correlation between lymph node metastasis in urinary bladder cancer patients and poor prognosis has been observed [90]. Using immunohistochemical staining, CCR7 was found to be elevated in urinary bladder cancers, which significantly correlated with positive lymph node status, tumor grade and lower overall survival [90]. In vitro experiments determined that CCL21/CCR7 activation enhanced urinary bladder cancer cell migration/invasion; however, this behavior was reversible upon CCR7 inhibition. Similar to what has been observed in primary T cells [91], migration of bladder cancer cells was dependent on activation of the ERK1/2 signaling rather than the PI3K/AKT pathway [90].

### 3.1. Gynecologic Cancers

Cervical cancer is the most common type of gynecological malignancy, being the fourth most common cancer in women, with most of these cancers associated with human papillomavirus infection [92]. Cervical squamous cell carcinomas had significantly elevated CCR7 expression linked to a more invasive and larger tumor size, as well as vaginal invasion and lymph node metastasis [93]. CCL19 has also been shown to be overexpressed in cervical cancer tissue and cell lines with siRNA-induced a reduction in CCL19 inhibiting cervical cancer cell proliferation and increased levels of apoptosis, suggesting that CCL19 via CCR7 activation is a driving force of cervical cancer progression [94]. Both CCR7 and CXCR4 expression were independent prognostic factors for reduced survival from ovarian cancer. Like some other tumors, CCR7 expression was mainly cytoplasmic and rarely nuclear localization, which occurred when there was no lymphatic involvement [93]. A further study confirmed the frequent CCR7 expression in ovarian carcinoma tissues and association with advanced tumor stage and lymph node metastasis. Furthermore, in vitro studies using human ovarian epithelial cancer cells, SKOV-3, indicated that CCR7 expression was elevated under hypoxic conditions and activation by CCL21 increased EMT development and ovarian squamous carcinoma cell invasion [95].

In contrast to earlier studies, analysis of differential gene expression suggested an important role of CCR7 in protection from cervical cancer. In a screen of 1367 differentially expressed genes in cervical cancer in The Cancer Genome Atlas (TCGA) database, 79 prognostic differentially expressed genes were found, and four of these genes, including CCR7, were further validated in the Gene Expression Omnibus database [96]. High expression of these four genes—CCR7, programmed cell death-1 (PD-1), ZAP70 and CD28—was linked to a better 5 year overall survival [97]. Further analysis of TCGA and protein–protein interactions supported a positive correlation between CCR7 expression in cervical squamous carcinoma cells and patient survival, which was linked to a predominant augmentation of immune-related pathways, as opposed to a more metabolic pathway described for the low CCR7 expression group [98]. Another recent analysis of TCGA database suggested an immune gene-related prognostic model for cervical cancer that includes CCR7, along with CD3d, CD3e, β2 integrin, family with sequence similarity 133 member A and p53 for forecasting survival and immune responses for cervical cancer patients [99]. The apparent contradiction between CCR7 increasing lymphatic metastasis and reducing survival for cervical carcinomas, yet being protective when analyzed within large differential gene expression analysis likely reflects the fact that the latter includes the tumor environment and thus reflects the positive effects of CCR7-expressing immune cells on tumor regression.

The Crk-like adapter protein (CrkL) can be induced by CCL19/CCR7 activation in the process of ovarian epithelial carcinogenesis [100]. Both CCR7 and CrkL are overexpressed in ovarian epithelial carcinoma cells lines and tissue samples, correlating with higher-stage, lymph node metastasis and activation of the EMT markers and reduced overall survival [100]. In SKOV-3 cells, CrkL knockdown attenuated CCL19/CCR7-activated EMT progression compared to control cells, operating through the ERK signaling pathway [100].

### 3.2. Prostate Cancer

As previously discussed, many cancers frequently metastasize to lymphoid tissue, for which CCR7 is often the perpetrator. Prostate cancer can invade the lymph nodes, but less frequently than primary target, bone. A review of over 30 years’ worth of case reports found 153 patients presenting with lymphadenopathy although linkage to chemokines/chemokine receptors was unknown [101]. In a case study, the same group showed intense antibody staining of CCR7 in prostate cancer tissue, which was the first time that high CCR7 had been reported and that likely explained the positive lymph node status of the patient [101]. A later report of another patient with lymph node involvement also showed high CCR7 expression, along with the B-cell marker, CD20 [102]. Despite the relatively modest incidence of CCR7 effects on the progression of prostate cancer, it was noted in vitro that siRNA against CCR7, in PC-3 prostate adenocarcinoma cells, not only silenced CCR7 but also inhibited VEGF and MMP-9 protein expression [103]. In the same PC-3 xenograft mouse model, CCR7 knockdown decreased prostate cancer tumor volumes compared to controls, suggesting that the CCR7 pathway affects tumor proliferation [103]. Further studies have investigated CCR7 effects on prostate cancer cell growth. A CCR7-expressing vector was transfected into PC-3 cells, which elevated expression of Notch1, p-MAPK, p-p65, MMP-9, N-cadherin and Snail, which are features of EMT and as expected was indicated by enhanced migration and invasive cell characteristics [104]. Prostate cancer cell lines were used to show that low expression of TNF-α induced CCR7 expression. Furthermore, CCL21 activation of CCR7 promoted the migration of prostate cells via phosphorylation of p38 MAPK, suggesting a potential pathway for lymph node metastasis of prostate adenocarcinoma [105]. Overall, the data supports mechanisms for CCR7-mediated prostate cancer lymphoid migration, although these pathways are likely not active in many prostate cancer patients.

As with many cancers, studies in genitourinary cancers yield inconsistent results regarding the roles of CCR7 in the progression of the disease (Table 2). For instance, in bladder and prostate cancers, expression of CCR7 was linked to elevations in MMPs and more aggressive tumors. Downregulation of CCR7 in animal models limited tumor aggression. In contrast, CCR7 expression in cervical cancers could be linked to improved or reduced overall survival, dependent upon the study. In some cases, when tumors co-expressed CCR7 with other proteins such as PD-1, ZAP-70 and CD28, patient survival rates were improved, when compared to patients who did not co-express these markers. Since PD-1, ZAP-70, CD28 and CCR7 are all normally expressed in immune cells, the co-expression of these markers may reflect a tumor environment that promotes anti-tumor immunity. It will be important in future studies to confirm that the CCR7 expressed is indeed inside of the tumor cells.

## 4. The Roles of CCR7 in Gastrointestinal Cancers

### 4.1. Colorectal Cancer

Currently, the lifetime risk of developing colorectal cancer is 4.3% for men and 4.0% for women in the United States [35]. The link between CCR7 expression and colorectal cancer has shown variable results. Specifically, there is a potential role for CCR7 in colorectal cancer progression based on the overexpression of CCR7 ligand CCL21 observed in the inflammatory bowel disease, ulcerative colitis [106,107,108]. In ulcerative colitis, which was associated with elevated levels of CCR7, the receptor was postulated as an inflammatory marker of disease progression to colorectal cancer [109]. Lymph node status correlated with CCR7 expression by immunohistochemical analysis of 99 colorectal patients at various clinical stages of tumor progression, although, overall, 5 year survival was significantly lower for CCR7-positive tumors [110]. In an in vitro/in vivo mouse experiment, CCR7 in SW620 human colon carcinoma cells was knocked down in cell culture using anti-CCR7 siRNA. When these cells were injected into the lumbar region of athymic nude Balb/c mice, cancer invasion and metastasis to lymph nodes was reduced, when compared to control cells with 4-fold higher levels of CCR7 expression [111]. Further analysis of colorectal cancers revealed that CCR7 expression was highly variable, although rarely absent from patient tumor specimens. A recent article confirmed that colorectal cancers expressed increased levels of CCR7, which correlated with tumor size and poorer overall survival; notably, this elevated expression was commonly associated with primary tumors of the rectum [112]. These results demonstrated a correlation between CCR7 expression and colorectal lymph node metastasis. These results, however, are not universal. In a related study, CCR7 expression was observed to be highly variable in 96 colorectal carcinoma patients and although CXCR4 expression was associated with lymph node metastasis, in this study, there was no such correlation for CCR7 [113]. 

CCR7 has been found mostly in the cytoplasm of cancers when evaluated by immunohistochemical analysis. Indeed, in the above-mentioned study where CCR7 expression did not correlate with colorectal lymph node metastasis, CCR7 staining was mainly cytoplasmic [113]. Like some of the other studies reviewed in breast cancer, it is unfortunate that the authors did not validate the CCR7 Western blots used in this study with negative controls that lacked CCR7 expression. In contrast to the ERK1/2 phosphorylation response to CCR7/CCL19 activation observed in immune cells [114], the colon cancer cells did not appear to express functional CCR7, since the cells failed to activate signaling pathways via CCL19 or CCL21 to ERK1/2 [113]. A second study reported that membrane staining of CCR7 was not found in colorectal cancer cell lines or primary tumor tissue samples [115]. In this study, while DNA mutations were not seen, most samples contained a truncated CCR7 mRNA, suggesting alternative splicing or possibly post-transcriptional mRNA changes. These CCR7 variants coded for truncated signal peptides that prevented this form of CCR7 from embedding in the cell membrane or responding to CCR7 ligands. The cellular function of the truncated form of CCR7 was not determined although it was hypothesized to confer a growth and/or survival advantage to the colorectal cancer cells [115]. In these studies, CCL21 expression levels were significantly reduced in colorectal tissue, when compared to non-cancerous tissues from the same patient; the significance of which was unclear [116]. Moreover, expression of the other CCR7 ligand, CCL19, is also attenuated in colorectal tissue compared to normal tissue; indeed, colorectal cancer patients with elevated CCL19 had statistically increased survival compared to CCL19-negative patients [117]. This response may, at least in part, be due to the CCL19-mediated recruitment of immune cells, which may induce the host immune response against the colon cancer. Moreover, CCL19 may also mediate inhibition of colorectal carcinoma angiogenesis via inhibition of the VEGF-A pathway [118]. Overall, the data confirms that the relationship between CCR7, its ligands and colorectal cancer progression and metastasis, particularly to lymph nodes, is complex depending, at least in part, on the locality of the cancer, the cellular location of CCR7 and the effect of truncated versions of the receptor.

Like what was observed in breast cancer, expression of CCR7 in colon cancer can elevate the EMT markers. Specifically, MMP-9 is expressed in CCR7-expressing colon cancers, with a downstream response of lymph node metastasis. In the human colorectal carcinoma cell line, SW480, CCR7 knockdown using shRNA led to reduced MMP-9 levels that, when tested in a xenograft mouse model, lowered colon cancer metastasis and increased animal survival when compared to CCR7-expressing tumor cells [119]. It was inferred that the reduced CCR7 levels led to the inability of colorectal cells to attach and grow in lymph nodes [119]. Like breast cancer, CCR7 was upregulated by COX-2 activity in colon cancer as well, although the correlation with cancer progression was undetermined [120].

There is some controversy regarding whether CCL21 can, in addition to CCR7, bind another chemokine receptor, CXCR3. An early mouse study indicated that CCL21 can bind CXCR3 [121]. Subsequently, it was suggested that human CCL21 does not bind to human CXCR3 but mouse CCL21 can bind to mouse CXCR3 with moderate affinity (Jenh, 1998). In one study, CXCR3 was reported to be highly expressed in human colon cancer epithelium in approximately a third of patient samples, but not in normal colon epithelial cells. The high levels of CXCR3 expression led to increased lymph node metastasis and worsened outcomes in patients, when compared to non-CXCR3 expressors. Surprisingly, CCR7 expression was not linked to lymph node metastases or patient survival [122].

Cancer treatments or what changes such treatments would have on CCR7 functions is not covered in this review; however, it is noteworthy that CCR7 appears to play a significant role in cetuximab resistance in colorectal patients. Cetuximab is an anti-epidermal growth factor receptor (EGFR) monoclonal antibody used as a single agent in patients with *KRAS* metastatic colorectal cancer, for which many patients acquire resistance. EGFR is highly expressed in such tumors and co-localizes with CCR7, only in patients resistant to cetuximab [123]. Further in vitro analysis demonstrated that CCL21 addition reduced the rate of cetuximab resistance and promoted EMT transformation. In contrast using an antibody to neutralize CCR7 and a p-AKT inhibitor reversed the EMT transformation. Thus, the combination of the CCR7 function-blocking antibody along with a p-AKT antagonist may serve as a platform for a therapeutic against *KRAS*-expressing metastatic colorectal cancer [123].

### 4.2. Esophageal Cancer

Esophageal carcinoma is typically highly aggressive, often with lymph node metastasis and vascular invasion and a 5 year survival rate of between 20 and 30% [124]. CCR7 mRNA was detected in 9/20 esophageal squamous cell carcinoma cell lines with CCL21 activating cell migration and pseudopodia formation. High CCR7 expression in esophageal squamous cell carcinoma tissue samples correlated with lymph node metastasis, higher tumor stage and decreased survival time [124]. A similarly high CCR7 mRNA level in esophageal cancer cells from tissues correlating with lymph node metastasis was observed, although, overall, CCR7 mRNA levels in primary esophageal tumor cells did not show such a correlation, likely due to the presence of CCR7-positive infiltrating lymphocytes within the lymph nodes [125]. CCR7 mRNA was an independent predictor of a high percentage of esophageal recurrences when measured as 3 year survival and had a worse survival prognosis for patients with co-expression of CCR7 mRNA and VEGF-C mRNA when compared to non-expressors [126]. CCR7 was also frequently co-expressed with MUC1, the gene for mucin-1, in esophageal squamous carcinomas with both being linked to lymph node metastasis and poor prognoses. Furthermore, MUC1 inhibition suppressed cancer cell invasion induced by CCL21 [127]. In a mouse model of esophageal squamous cell carcinoma, CCL21 activation of CCR7-expressing cells increased cell adhesion that had a higher lymph node metastatic behavior [128]. Overall, there is a strong link between CCR7 expression and esophageal cancer metastasis to the lymph nodes with associated rapid cancer progression and poor survival.

### 4.3. Gastric Cancers

Worldwide, gastric cancers are the fourth most common cancers in men and the fifth most common in women. Approximately one million new cases are diagnosed every year with more than 70% occurring in developing countries [129]. An early study of CCR7 expression in gastric cancer analyzed 10 human gastric cancer cell lines and 43 gastric cancer tissues by RT-PCR and an additional 307 gastric cancer tissues by immunohistochemistry [130]. CCR7 was expressed in all gastric cancer cell lines and 84% of gastric cancer tissues by RT-PCR and 22.5% by immunohistochemistry [130]. CCR7 protein levels were higher in differentiated vs. undifferentiated gastric cancer subtypes and CCR7 expression was not associated with lymph node metastasis. Moreover, patients with CCR7+ gastric cancers had a better prognosis than patients with CCR7- gastric cancer [130]. While these results are interesting, although the investigators found by RT-PCR that ~84% of patient samples were CCR7(+), unfortunately, the anti-CCR7 antibody used in the IHC only stained 23% of their tissues, making the results difficult to interpret. In a small study, four of six gastric carcinoma cell lines expressed CCR7 and were able to migrate in response to CCL21. Clinical gastric cancer specimens had a similar propensity for CCR7 expression (42/64, 66%), which correlated with lymph node metastasis [131]. Another study reported lower levels of CCR7 expression in resected gastric carcinoma cells (30/93, 32%), which again correlated with lymph node migration [132]. A more recent study concurred that gastric cancer expresses CCR7 at high levels, with ~70% CCR7 expression from 133 patient samples [133]. CCR7 expression was linked to the presence of intratumoral FOXP3+ Treg cells, suggesting that the gastric cancer milieu favored tumor survival and CCR7-mediated lymphatic invasion [133]. 

Infection with *Helicobacter pylori* bacteria causes chronic gastric inflammation and significantly increases the risk of developing gastric ulcers and gastric cancer. Infection with *H. pylori* is the strongest known risk factor for gastric cancer [134]. Two studies investigated the effects of *H. pylori* on CCR7 levels in gastric epithelial cells. In the first study, CCR7 expression was limited to the gastric epithelium of all patients tested [135]; however, receptor staining was stronger in *H. pylori*-infected gastric cells, which included gastric carcinoma. In this study, it was determined that CCR7 expression was regulated by *H. pylori* [135]. The second study found that neoplastic transformation of *H. pylori*-linked gastritis to mucosa-associated lymphoid tissue (MALT) lymphoma and to gastric extranodal large B-cell lymphoma included upregulation of CCR7 and other chemokine receptors, although non-cancer gastric tissue samples did not express CCR7 [136]. Overall, the data supports a role for CCR7 in *H. pylori*-linked gastritis and gastric cancer progression.

VEGF-C and CCR7 were expressed in approximately half of gastric cancer tissue specimens and co-expression of VEGF-C and CCR7 was a strong predictor of lymph node metastasis [137]. Another study of 82 gastric cancers found that VEGF-C, VEGF-D and CCR7 were present in 88%, 63% and 67% of cases, respectively [138]. All three markers predicted lymphatic invasion of the primary gastric tumor but none predicted lymph node metastasis, which was somewhat surprising considering CCR7 ligand expression in the lymph nodes [138]. As mentioned previously, miR-let-7a can modulate CCR7 expression and this was also seen in gastric cancers, where high CCR7 expression was associated with reduced levels of miR-let-7a, most likely due to the low expression of Dicer 1 that is required to produce the microRNA [139].

Under hypoxic conditions that frequently occur in solid tumors, HIF-1α can be released. In gastric cancers, HIF-1α can upregulate CCR7 along with increasing COX-2 production and expression of MMP which are associated with EMT and poor survival [140]. Analysis of 122 patients with gastric cancer revealed that EMT in gastric cancers was mediated, at least in part, by CCR7. Upregulation of CCR7 in tumors enhanced TGF-β1-induced EMT and could be inhibited by a CCR7 neutralizing antibody [141]. A second study reported that CCR7 expression in gastric carcinomas was closely linked to expression of the transcription factor, Snail, which represses E-cadherin, thereby promoting EMT. In addition, CCR7/Snail upregulation led to increased levels of the EMT markers, p-ERK, p-AKT and MMP-9 and sped up the G_1_/S phases of the cell cycle when the human gastric cancer cell line, MGC803, was incubated with CCL19 [142]. Taken together, these results suggest a key role for CCR7 in EMT progression of gastric cancers.

In contrast to previously mentioned results, a meta-analysis suggested that CCR7 can also be a poor prognostic marker for gastric cancer progression. In this study, a meta-analysis was performed on 15 eligible studies, totaling 1697 patients, to assess the gastric cancer risk of CCR7. The pooled hazard ratios indicated a statistically significant risk of a lower 5 year overall survival rate for CCR7+ vs. CCR7− gastric cancers (HR = 0.46, 95% CI 0.31–0.70. *p* < 0.001). Other statistically significant end points for CCR7+ vs. CCR7− gastric cancers included deeper tumor invasion, advanced stage, vascular invasion, lymph node metastasis and lymphatic invasion [143]. Even though the current epidemiological data indicates that CCR7 activation is an undesirable parameter for gastric cancer, results are not unequivocal. 

### 4.4. Pancreatic Cancer

Pancreatic cancer is the fifth leading cause of death from cancer worldwide and is a highly aggressive malignancy with a five-year survival rate of less than 5% [144]. CCL21 levels were low and CCR7 levels high in pancreatic cancer tissue compared to normal pancreas [145]. This apparent contradiction between receptor and ligand expression related to vessel density localized to the pancreatic cancer, such that CCL21 expression was linked to microvessel density but not microlymphatic vessel density, whereas for CCR7 expression, effects were reversed. Unfortunately, CCL19 expression was not evaluated in this study; however, the available data suggests that CCR7, presumably activated by CCL19, allows for pancreatic cancer metastasis to lymphoid tissue. Alternatively, it is possible that CCL21 expression was not detected in the pancreatic cancer tissue because the ligand did not bind the CCL21 antibody. It has been demonstrated that the C-terminal tail of CCL21 can be cleaved to produce a truncated soluble form of CCL21 that is often not detected by antibodies to full-length CCL21 (Bastow, 2021). PT45P1 cell line, derived from a grade III pancreatic cancer transfected with CCR7 and orthotopically transplanted into nude mice, gave rise to significantly larger tumors and a higher frequency of lymph vessel invasion than mock transfected cells [146]. Analysis of microdissected pancreatic cancer samples found that expression of CCR7 was associated with lymph node metastasis and tumors that lacked CCR7 had low rates of lymphoid tissue invasion [146]. A second study reported a similar positive correlation between CCR7 levels and lymph node metastasis in pancreatic cancer tissue from patients [147].

As previously discussed for several cancers, CCR7 is associated with EMT, and this is further consolidated in pancreatic cancer. Transcription factor, Twist, promoted EMT in pancreatic adenocarcinoma, leading to tumor progression, and was expressed in 72% of patient samples and aligned with tumor stage and lymph node metastasis [148]. Stimulation of the CCR7-expressing pancreatic adenocarcinoma-derived cell line, PANC1, with CCL19 led to enhanced expression of p-ERK, p-AKT, N-cadherin and MMP-9, markers of EMT progression, further implicating CCR7 in the metastatic progression of pancreatic adenocarcinomas [148]. CCR7/CCL21 activation and its role in EMT using different pancreatic adenocarcinoma cell lines and resected tissue were investigated. CCR7 levels were significantly increased in CD133+ pancreatic cancer stem-like cells compared to CD133− cancer cells and normal tissue and lymph nodes [149]. CCR7/CCL21 promoted survival and metastasis of the CD133+ pancreatic cancer cells via modulation of the ERK/NF-κB pathway [149]. PANC1 cells were transduced with a lentiviral vector expressing CCL21, which promoted MMP-9 expression, like CCL19 described above. In addition, DNA microarray data identified several CCL21-mediated genes that were upregulated including ATM and BRCA1, whereas downregulation of the pro-apoptotic gene, CASP8 was noted [150]. These results suggest a CCL21-induced pro-survival response of pancreatic cancer cells; however, downregulation of AKT1, FOS and JUN and angiogenic cytokines indicates the anti-proliferation effects of CCL21, which somewhat complicates the mechanism of action [150].

A common feature of pancreatic adenocarcinoma is progressive pain as the tumor grows. Sensory neurons can produce CCL21 in pancreatic adenocarcinoma to enhance cell migration in patients and orthotopic tumors in mice. When CCL21 was inhibited in mouse studies, significant reductions in nociceptive hypersensitivity and nerve fiber hypertrophy were observed along with improved behavioral events, although tumor infiltration was not affected. The results suggested that CCL21 promotion of pancreatic cancer cell growth towards sensory neurons was important for pain development [151].

As observed with breast and gastrointestinal cancers, the cancers of the gastrointestinal tract have inconsistent responses to overexpression of CCR7 (Table 3). Elevated expression of CCR7 or its ligand CCL19 in colorectal or gastric cancer correlated with lymph node metastases, and an improvement in overall survival [110,117,130]. However, these results were variable. In colorectal cancer, where CCR7 does not correlate with survival, the receptor is found within the cytoplasm, where it cannot signal in response to ligands [113]. This could be due to CCR7 promotion of EMT, leading to cells that could not attach to lymphoid organs [119]. Interestingly, when CCR7 was co-expressed with CXCR3, the presence of CXCR3 negated the survival benefit of CCR7. In contrast, when CCR7 was expressed alone or in combination with elevated MUC1 or CCL21 in esophageal cancers, the presence of CXCR3 worsened the prognosis [126,127,128]. However, VEGF-C co-expression with CCR7 was a strong predictor of lymph node metastasis [137], and a meta-analysis of 15 studies found that CCR7 is a marker of a poor prognosis [143]. In pancreatic tumors, however, the results were more consistent, demonstrating a role for CCR7 in lymph node metastasis [147], EMT [148] and progressive pain [151]. Clearly, CCR7 has distinct effects in different gastric tumors. Further studies should be conducted to help define mechanisms that promote the improved CCR7-related survival seen in certain patients.

## 5. Head and Neck Cancers

### 5.1. Oral

Oral squamous cell carcinoma is the most frequently occurring oral cavity cancer, associated with substantial local invasion and metastasis to the cervical lymph nodes [152,153]. Such lymph node metastasis suggests a potential role for CCR7. Among cases of oral and oropharyngeal squamous carcinoma cases, approximately 65% were positive for CCR7, which correlated with tumor progression, large lymph node metastases and reduced survival; normal oral mucosa was negative for CCR7 staining [154,155]. An early study used Plt mice that exhibit reduced CCR7 responses compared to wild-type mice with fully functional CCL19 and CCL21 genes [156]. Murine oral squamous cell carcinoma cell line, B7E3, implanted in syngeneic Balb/c mice had a significantly higher rate of tumor growth and cervical lymph node metastasis compared to plt littermates, which, at least in part, could be overcome by overexpressing CCR7 in plt mice to counter the plt CCR7-activation defect [157]. This is curious, given that in the absence of CCR7 ligands, CCL19 and CCL21-ser, it was unclear how the CCR7-expressing cells could become activated. The above results contrast with a study that reported similar levels of CCR7, along with CCL19 and CCL21 mRNA in both oral squamous carcinoma and normal oral mucosa, which led the authors to conclude that the CCR7/CCL21/CCL19 pathway was likely not responsible for the observed cervical lymph node metastasis and further suggested that CXCR4/CXCL12 axis is primarily responsible instead [158]. Generally, the results suggest a key role for CCR7 in oral squamous carcinoma lymph node metastasis, although other pathways, such as CXCR4/CXCL12, might also be involved.

Tongue squamous cell carcinoma is a common (25 to 40%) type of all oral cancer. High CCR7 expression significantly correlated with cervical lymph node metastasis and histological grade of tongue squamous cell carcinoma [159]. Using the tongue squamous cell carcinoma cell line, SCC4, CCR7 activation promoted a more aggressive phenotype, whereas CCR7 inhibition reduced cell migration and invasion without affecting cell growth or survival [159]. Furthermore, when SCC4 tumors were grown in a nude mouse model, CCR7 knockdown reduced tumor growth, inhibited cervical lymph node metastasis and extended survival. A correlation between CCR7 activity and lymphatic vascular density was noted, as was expression of CCR7 and VEGF-C [159]. Further, a significant association between CCR7, VEGF-C, and VEGFR-3 expression and lymph node metastasis were observed [160]. In this study, CCR7 tissue immunostaining was high in tongue cancer and was significantly associated with male tongue cancer patients compared to females. Interestingly, there was no association between elevated CCR7 levels and tongue cancer prognosis [160]. Using paraffin-embedded tongue squamous carcinoma tissue samples, a higher expression of CCR7, along with CCR5 were independent biomarkers of poor prognosis and shorter disease-free survival of patients [161]. 

Abnormal expression of long non-coding RNAs has been noted in several cancers including tongue squamous carcinoma [162]. A study found that long non-coding RNA urothelial cancer-associated 1 (UCA1) was upregulated in conjunction with CCR7 in tongue squamous carcinoma cells and, if either were silenced, there was a reduction in cell proliferation, migration/invasion and glycolytic metabolism. The authors speculated that UCA1 might function as an oncogene in tongue squamous carcinoma by regulating the CCR7 pathway [163]. Another oral cancer, squamous carcinoma of the tonsils, showed that at high CCR7 levels, patients had a significant (*p* < 0.001) increase in cervical node metastasis, relapse-free (*p* = 0.0175), overall and disease-free survival rates *p* = 0.0062 [164]. In general, high levels of CCR7 predict poor prognosis for patients with oral cancers. It may be worthwhile to examine CCR7 antagonists in the future as potential treatments for patients to prevent further proliferation and additional metastasis of tumors.

### 5.2. Non-Oral

Head and neck carcinoma is the sixth most common aggressive cancer in the world, and 90% of these malignancies are squamous cell carcinomas [165]. Overall, the 5 year survival rate for head and neck squamous carcinoma patients is poor (30–40%), primarily due to cervical lymph node metastasis [166]. Several studies have investigated the expression and role of CCR7 in general head and neck cancer tissues and cell lines. When 9 head and neck squamous carcinomas cell lines and 25 tissue samples were tested by semi-quantitative RT-PCR, all samples were positive for CCR7 with high CCR7 mRNA correlating with poorly differentiated tumors and lymph node metastasis [167]. CCR7 was linked to local recurrence, being male and smoking, which were risk factors for poor prognosis, suggesting a role for CCR7 in cancer progression [168,169]. Tissue microarray analysis from 50 patients with head and neck cancer showed that 40% of these cancers expressed the transcription factor, Twist, which is known to be activated in several metastatic tumors resulting in the reduction of E-cadherin and an EMT phenotype associated with reduced differentiation status and lymph node metastasis [170]. Twist expression significantly correlated with CCR7 and CXCR4 levels, leading the authors to speculate that Twist might regulate CXCR4 and CCR7 expression, although it seems more likely to be the other way round since, at least for pancreatic ductal adenocarcinoma, it was shown that CCR7 regulates Twist [148].

Epithelial nasopharyngeal carcinoma frequently metastasizes to bone, liver and lymph nodes [171]. Immunohistochemical staining of patient cancer samples revealed heterogeneous expression of CCR7, CXCR4 and CXCR6 with low expression in most primary tumors and strong chemokine receptor expression in metastatic lesions to the liver [172]. Serum levels of CCR7 appeared to be a relevant marker for patients with locally advanced nasopharyngeal cancer since a higher concentration of CCR7 was a good predictor for a locally advanced tumor and poor prognosis [173]. Salivary adenoid cystic carcinoma is the second most common malignancy of salivary glands. Chemokine receptor analysis of salivary adenoid cystic carcinoma cell lines, SACC-83 and SACC-LM, SACC cell lines with high levels of metastasis to the lungs showed that expression levels for all analyzed chemokine receptors, including CCR7, was higher in the SACC-83 cell line compared to SACC-LM, suggesting that these receptors were not significant contributors to lung metastasis [174]. This was in line with our observation that in a murine model, breast cancers that were metastatic to the lung also had reduced lung metastases in the presence of CCR7 [33]. Future studies to examine the factors in the lung that oppose the proliferation of CCR7(+) tumors may reveal novel platforms for treating CCR7-expressing tumors in other sites within the body.

Key regulators of cell adhesion are integrins, transmembrane glycoproteins, which mediate cell–cell and cell–matrix interaction and can facilitate metastatic progression. CCL19 activation of CCR7 in the metastatic squamous head and neck carcinoma cell line, PCI-37B, which expresses CCR7, led to upregulation of β3 integrins and enhanced migration and reorganization of actin cytoskeleton. These effects were blocked by the αvβ3 integrin-specific inhibitor, IS201 and were induced by αvβ3 integrin phosphorylation [166,175]. Overall, the data suggested that CCR7 regulated cell adhesion in metastatic squamous head and neck carcinoma cells via αvβ3 integrin [175]. Src, a non-receptor protein tyrosine kinase, is activated in several cancers and promotes integrin functions [176]. PCI-37B cells incubated with CCL19 upregulated p-Src along with p-Pyk2 and p-Paxillin and cells showed more invasive and migratory characteristics. The Src inhibitor, PP2, downregulated all three proteins and reversed cell invasive and migratory phenotypes [177]. These data support a role for Src/integrins in CCL19/CCR7 head and neck squamous carcinoma progression.

Further investigations of the CCR7 signaling events in head and neck squamous cell carcinomas used PCI head and neck squamous carcinoma cell lines. CCR7 bound to CCL21 or CCL19 activates phosphoinositide-3 kinase (PI3K) [178], which in turn activates Akt to facilitate pro-survival responses [179]. Using two CCR7(+) lymph node metastasis-derived squamous cell carcinoma cell lines, autologous CCL19 induced the phosphorylation of mammalian target of rapamycin (mTOR). This was inhibited by blocking the CCR7-PI3K pathway, resulting in apoptosis and cell-cycle arrest [180]. Thus, CCR7 is thought to be a key modulator of head and neck squamous carcinoma survival [180]. In a follow-up study, the group determined that stimulation of CCR7 by CCL19 induced JAK2/STAT3 phosphorylation, which was blocked by anti-CCR7 monoclonal antibodies [181]. Furthermore, the JAK2/STAT3 pathway mediated CCR7-induced cell migration and invasion speed linked to lymph node metastasis via EMT [181]. In a third study, these authors reported that CCL19 stimulation of CCR7 induced ERK1/2 and JNK phosphorylation but had no effect on p38, effects also associated with EMT activation pathways [182]. Additionally, CCL19 induced the Rho GTPase, Cdc42, localization to the cell membrane and actin polymerization in migrating head and neck squamous carcinoma cells. CCR7 and PI3K inhibitors prevented cell migration, Rac activation and actin polymerization in the presence of CCL19, as did knockdown of Cdc42 by small interfering RNA, suggesting an involvement of Cdc42 in the CCR7-PI3K pathway [183].

Inflammatory mediators are commonly produced by head and neck squamous cell carcinomas in response to inflammation and tissue damage. In vitro studies using PCI-6A, PCI-15A and PCI-37A head and neck squamous carcinoma cell lines determined that human β-defensins, small antimicrobial peptides secreted by epithelial cells, induced cell membrane CCR7 expression, promoting cell migration towards CCL19 [184]. Inhibition of NF-kB, a known regulator of CCR7 expression, lowered CCR7 levels, cell survival and migratory behavior [184]. A follow-up study confirmed the co-expression of NF-kB and CCR7 in head and neck squamous cell carcinoma cells; however, NF-kB inhibition only partially reduced CCR7 levels since AP-1 transcription factor also controlled CCR7 expression in these cells [185]. In PCI-37B cells, CCL19 induced activation of protein kinase C alpha (PKCα), which was abrogated by an anti-CCR7 monoclonal antibody [186]. PKCα inhibition reduced NF-kB activity induced by CCL19. Notably, while immunohistochemical analysis of head and neck squamous carcinoma tissue confirmed high expression of CCR7 and PKCα, neither marker was seen in adjacent normal tissue [186]. Overall, this data supports a signaling pathway involving CCR7, NF-kB and PKCα in promoting head and neck squamous carcinoma progression and lymph node metastasis.

Like its reported role in bladder cancer, a study found that MMP-9 is upregulated by CCR7 in the PCI-37B head and neck squamous carcinoma cell line. CCL19/CCR7 upregulated MMP-9 protein with concomitant cell chemotaxis and reorganization of the actin cytoskeleton. These effects were prevented by the MMP-9 inhibitor, SB-3CT. Both CCR7 and MMP-9 expression were weak in normal human mucosal tissue [187]. Related to actin cytoskeleton, using PCI-37B cells inhibition of Ras homolog family member A (RhoA), a small GTPase, attenuated the cancer cell migration and invasive properties induced by CCL19; conversely, CCL19 incubation activated RhoA, the non-receptor proline-rich tyrosine kinase, Pyk2 and increased cofilin activity and actin polymerization, effects that were prevented by anti-CCR7 monoclonal antibodies [188]. Thus, CCR7 acting via RhoA/Pyk2/cofilin/actin promotes migration and invasive behavior of head and neck squamous carcinomas. The role of Pyk2 in head and neck squamous carcinomas was further investigated using a stable Pyk2-related non-kinase (PRNK)-expressing PCI-37B cell line, which downregulated Pyk2 activity and inhibited CCL19-induced CCR7 effects including reduced E-cadherin and vimentin expression. This cell line had low viability, increased apoptosis and low migratory abilities. When grown in nude mice, the resultant tumors were slow growing compared to control tumors with normal Pyk2 and CCR7 responses [189]. Since E-cadherin and vimentin expression have been linked to EMT and a metastatic phenotype in head and neck squamous carcinomas [190], the results suggested that CCR7-induced metastasis in these tumors is Pyk2 dependent [189].

Solid cancers including head and neck squamous cell carcinomas often develop hypoxic conditions as the tumor develops and such tumor environments can promote metastasis [191,192]. CCR7 expression was investigated under normoxic or hypoxic conditions in several head and neck cancer cell lines growing in vitro as monolayers or 3D spheroids, or in vivo after xenografting into Balb/c mice. Each model showed elevated levels of CCR7 expression under hypoxia. Cancer tissue showed correlative responses of CCR7 and HIF-1α, along with a more malignant phenotype [193].

MicroRNAs have different functions in malignancies, depending on the cancer type. One such microRNA, hsa-miR-125a-5p, appears to have both oncogenic and tumor-suppressive characteristics. In oral squamous cell carcinoma cells, hsa-miR-125a-5p levels were attenuated in tumor tissue relative to normal tissue; however, for head and neck squamous carcinoma cells, the correlation is not clear. For low tumor stage, hsa-miR-125a-5p levels were elevated relative to normal tissue but in higher-grade tumors the relative levels of hsa-miR-125a-5p decreased. Based on this data, it is surprising that hsa-miR-125a-5p expression in head and neck squamous carcinoma tissue was linked to shorter patient survival [194]. Using PCI-37B cells, transfected hsa-miR-125a-5p upregulated CCR7 expression with associated enhanced cell proliferation, migration and invasion, which fits better with the patient survival results [194,195]. Similar results were observed for another microRNA, miR-1275, when transfected into PCI-37B cells, which also elevated CCR7, leading to more aggressive cancer cell characteristics [196]. Another microRNA, hsa-let-7e-5p, was investigated for its effects on head and neck squamous cells and CCR7 expression. Upon hsa-let-7e-5p transfection into PCI-37B cells, quantitative real-time PCR showed a significant reduction in CCR7 mRNA, with resultant decreased protein levels and reduced cell proliferation both in vitro and in a xenograft Balb/c mouse model [197]. An inhibitor of hsa-let-7e-5p increased CCR7 expression and elevated PCI-37B proliferation, migration and invasion [197]. These experiments indicate that hsa-let-7e-5p acts as a tumor suppressor by inhibiting CCR7 actions. Overall, it is likely that microRNAs play an important role in head and neck squamous carcinoma via diverse effects on CCR7 expression, and in turn, different effects on non-oral survival. In the future, it is likely that microRNAs will be used more frequently as anti-cancer agents.

### 5.3. Thyroid Cancer

Thyroid cancer is the most common endocrine malignancy in the United States. Papillary thyroid cancer is the most frequent form of thyroid cancer, making up ~80% of thyroid tumors [198]. Using real-time quantitative PCR, Sancho et al. found higher expression of CCR7 in papillary thyroid cancer and medullary thyroid cancer compared to follicular and poorly differentiated thyroid tumors. Within the papillary thyroid subtypes, CCR7 expression was 9-fold higher in the classic form compared to follicular variants, which correlated with lymph node metastasis [199]. CCL21 stimulation of CCR7 in the thyroid tumor cell line, TPC-1, promoted cell proliferation and migration via actin polymerization, increased β1-integrin expression and increased levels of MMP-2 and MMP-9, indicative of an invasive phenotype [199]. Immunohistochemical analysis of 88 papillary thyroid cancer specimens from 65 patients showed that samples having extrathyroidal extensions, angiolymphatic invasion or lymph node metastasis had elevated staining for CCR7 compared with those without the invasive characteristics [200]. A study of 30 patient samples of papillary thyroid cancer found that CCR7 was infrequently detected with 5–10% of cells being CCR7 positive. In contrast, CXCR4 was expressed in 90% of cells, and control cells did not express the chemokine receptors. Furthermore, there was no correlation between CCR7 expression and lymph node metastasis, although in this case there was a trend towards a correlation between CXCR4 expression in papillary thyroid cancer and lymph node metastasis [201]. The limited data suggests that CCR7 mediates lymph node metastasis of thyroid cancer, although CXCR4 may also have an important role.

CCR7 expression is often associated with more aggressive head and neck cancers with poor prognosis. Indicated pathways include CCR7-activated TWIST transcription factor and increased EMT, VEGF/CCR7-linked lymphatic invasion and αvβ3 integrin regulated increased cell adhesion in the presence of CCR7, which was p-Src dependent. In addition, CCR7-induced JAK2/STAT3 pathway mediated cell migration and invasion speed and lymph node metastasis. CCR7 also facilitated cancer cell survival via PI3K activation, phosphorylation of mTOR and Akt pro-survival responses (Table 4).

## 6. Tumors of Surface Epithelia

### 6.1. Lung Cancer

Worldwide, lung carcinoma is the primary cause of cancer-related death and non-small-cell lung cancer accounting for ~75% of lung cancers. Non-small-cell lung cancer (NSCLC) prognosis is poor, with <15% of patients surviving >5 years from the time of diagnosis [202]. Most non-small-cell lung cancers are classified as adenocarcinomas, with human A549 adenocarcinoma alveolar basal epithelial cells a commonly used cell line representative of this subtype of lung cancer. Murine CCL21 had no effect on the proliferation of A549 cells in vitro; however, when A549 cells were implanted into SCID mice, CCL21 reduced tumor size, which must be independent of T- or B-cell responses due to the immunocompromised nature of the mice [203]. A similar approach, in which A549 cells were orthotopically implanted into the lungs of athymic nude mice, led to lung nodules but no metastasis to lymph nodes; however, another non-small-cell lung cancer cell line, Lu-99, metastasized to the mediastinal lymph nodes, forming large nodules. Lu-99 cells expressed higher levels of CCR7 than A549 cells and metastasis to the lymph nodes in response to CCL21 was dependent on α4β1-integrin-mediated responses, with this integrin type present on Lu-99 cells but not A549 cells [204]. These results suggest that A549 cells might not be a good model for studying CCR7-mediated migration, although, as addressed below, CCR7 is expressed in A549 cells.

Despite reports suggesting that CCL21 does not affect A549 proliferation, another study reported CCL21 concentration-dependent A549 cell growth, associated with an increase in the G_2_M phase of the cell cycle, related to upregulation of cyclin A, cyclin B1 and cyclin-dependent kinase 1. CCL21-induced A549 growth was dependent on p-ERK levels, but not PI3K or Akt [205]. The same group showed that CCL21/CCR7 activation in A549 cells reduced apoptosis by upregulation of pro-survival bcl-2 and downregulation of pro-apoptotic bax and caspase-3, effects that were reversed by inhibiting CCR7 [206]. CCR7 inhibition in A549 cells attenuated TGF-β 1-induced EMT while inactivating NF-κB signaling and suppressing inflammatory responses [207]. A role for CCR7 in EMT of A549 cells was characterized after incubation with CCL19, which upregulated the transcription factor, SP1 and heparinase, an enzyme that cleaves heparan sulphate of extracellular matrix facilitating EMT and metastasis. Mechanistic studies suggested that SP1 bound to heparinase to activate enzyme activity in A549 cells [208]. In another study, CCL21 activation of CCR7 in A549 cells decreased expression of the epithelial marker, E-cadherin and upregulated the mesenchymal markers, Vimentin, Slug and ERK [209]. These results correlated with clinical samples from 50 lung carcinoma resections in which Vimentin and Slug levels were enhanced in samples with elevated CCR7 [209]. Combined, these results suggest that ligand-activated CCR7 enhances proliferation, survival and EMT of A549 cells. 

A previous link between CCR7 expression and VEGF-C expression has been noted for other cancers and was investigated in A549 cells. VEGF-C levels were downregulated in A549 cells by RNAi, which suppressed cell growth in vitro along with inhibiting CCR7-induced migration and invasion towards CCL21. When VEGF-C-inhibited A549 cells were injected into the tail vein of nude mice, suppression of tumor growth, angiogenesis and lymphangiogenesis was demonstrated with a concomitant reduction of the CCR7-dependent Akt, ERK1/2 and p38 pathways, as previously described for other cancers [210]. Another study using several NSCLC cell lines found that CCL21 activation of CCR7 enhanced expression of VEGF-D via ERK1/2 and Akt phosphorylation pathways [211]. Analysis of patient NSCLC tissue concluded that CCR7 and CCL21 levels correlated with VEGF-D expression, lymphatic vessel density, higher clinical stages, lymph node metastasis and decreased patient survival [211]. CCR7 was inhibited by siRNA in A549 cells and reduced cell migration and lymphoid metastasis was noted in both in vitro and in vivo athymic mouse studies along with attenuated expression of VEGF-C, VEGF-D and VEGF-R3 [212]. Overall, the data suggested that the CCR7/VEGF pathway is important for A549 lymphoid metastasis.

Solid tumors, including lung cancers, are often subject to hypoxic conditions as the tumor progresses. Ninety-four cases of NSCLC tissues were analyzed by immunohistochemistry, which determined that CCR7 expression positively correlated with levels of HIF-1α and HIF-2α [213]. Using the NSCLC cell line, BE1, that normally has low CCR7 expression, hypoxic conditions increased HIF-1α and HIF-2α and CCR7, like the tissue results. Furthermore, hypoxic conditions increased BE1 cell migration and invasion capacity in cell culture, which was ERK1/2 dependent [213]. The data suggested that hypoxia elevates CCR7 and promotes a metastatic phenotype of lung cancer. Although CCR7 is the primary chemokine receptor associated with lymph node metastasis, CXCR3, which may bind with moderate affinity to CCL21 in mice, has also been linked to lymph node chemotaxis in breast cancer and melanoma in a murine model [32,214]. In clinical lung adenocarcinoma tissue samples, both CCR7 and CXCR3 were expressed, with CXCR3 having a higher frequency, 90% vs. 65%; however, only CCR7 was associated with lymph node metastasis and not CXCR3 in these human tissues [215]. 

The importance of regulatory microRNAs in lung cancer control pathways has become increasingly evident. A study investigated two miRNAs—miR-335 and miR-let-7a, both of which could affect CCR7 expression. Tumor samples and normal tissue from 27 patients were analyzed and showed elevated CCR7 and CCL19 for patients with lymph node metastasis. These lymphoid metastatic patients also had higher expression of miR-335 and lower expression of miR-let-7a [216]. The results suggest that miR-335 upregulated CCR7 expression and miR-let-7a has opposite effects, such as its effects on CCR7 in breast cancer tissue [83]. The relevance of CCR7 to lung cancer was determined in a study looking for prognostic genes based on a screen that identified 158 potential candidates that were further analyzed by quantitative reverse transcriptase PCR on malignant samples from 147 NSCLC patients. Three genes were identified as prognostic: syntaxin 1A (STX1A), HIF-1α and CCR7 [217]. The link between HIF-1α and CCR7 in tumor lymph node metastasis was previously discussed, whereas the relevance of STX1A, a typically nervous system-specific protein, is unclear. The potential role of SNPs in CCR7-mediated lung cancer responses was investigated. Two CCR7 SNPs, *CCR7* rs3136685 and CCR7 rs17708087, were identified as having an elevated risk of NSCLC compared to the wild-type gene; both CCR7 SNPs are postulated to be histone epigenetic modifiers and may represent patients at particularly high risk of developing lung cancer [218].

Contrary to the role of CCR7 on lung cancer proliferation, survival and metastasis to the lymph nodes culminating in reduced survival, some publications suggest that CCR7 is beneficial in lung cancer rather than detrimental. In a study aimed at investigating the interactions between CrkL and non-receptor kinase, c-ABL and CCR7 in surgically resected lung adenocarcinoma from 120 patients, high CCR7 mRNA expression paralleled expressions of CrkL and c-ABL and was indicative of a better prognosis. Furthermore, elevated CCL19 levels were a good prognostic factor for lung adenocarcinoma patients [219]. Another study investigated CXCR4, CXCR5 and CCR7 expression and survival outcomes in patients with low-grade NSCLCs (T1N0M0) compared to local normal tissue. Enhanced expression levels of CXCR4, CXCR5 and CCR7 were found in tumor tissues compared to normal tissue. Moreover, 5 year disease-free survival and 5 year overall survival were significantly higher for lung cancer patients with positive expression for all three chemokine receptors compared to controls with CCR7 expression having the highest significance (*p* < 0.001 for both survival parameters) [220]. It is not obvious why these two studies buck the trend and suggest that CCR7 is the good guy; however, a recent bioinformatics study looked at various chemokine receptor expression patterns, including CCR7 expression, in relation to NSCLC stage compared to normal tissue and reported that CCR7 was expressed at all stages with higher expression in early stages that tails off as the tumor progresses [221]. These results suggest that the Yue, 2020 study focused on the highest CCR7-expressing stage and it is unknown whether CCR7 expression changed during tumor progression might better correlate with enhanced survival.

Overall, lung cancer metastasis to lymph nodes is dependent upon CCR7/ligand-mediated α4β1-integrin responses and upregulation of the transcription factor, SP1 with resultant activation of heparinase and breakdown of the extracellular matrix, promotion of EMT and metastasis. CCR7-induced lung cancer cell survival involved upregulation of pro-survival bcl-2 and downregulation of pro-apoptotic bax and caspase-3. Additionally, lymphangiogenic pathways were activated by CCR7-dependent Akt, ERK1/2 and p38 pathways and linked to VEGF family activation (Table 5).

**Table 5:** The roles of CCR7 in epithelial cancers.

Cancer	Observation	Reference
Lung	CCR7 in A549 reduces tumor size in the presence of CCL21	[203,204]
	Lu99 metastasized to LN via α4β1-integrins	[204]
	A549 cells grow when activated with CCL21 via upregulation of cyclin A, cyclin B1 and cyclin-dependent kinase 1	[205]
	CCL21/CCR7 reduced apoptosis via upregulation of Bcl-2 and downregulation of Bax/Caspase-3	[206]
	CCR7 inhibition in A549 induced EMT and suppressed inflammation	[207]
	CCR7 induced EMT via SP1 and heparinase to cleave ECM to facilitate EMT and metastasis	[208]
	CCL21 promoted E-cadherin and mesenchymal markers vimentin, Slug and ERK and correlated with clinical samples	[209]
	CCR7 mediated lymphangiogenesis via Akt, ERK1/2 and p38	
Skin	CCR7 may play a role in metastasis of non-melanoma skin cancers	[222]
	B16 transduced with CCR7/CCL21 enhanced tumorigenesis and increased metastasis to 50% of mice compared to 5% of controls	[223,224,225]
	A375 malignant melanoma migrate to CCL21 in vitro and in vivo	[31,223,225,226]
	CCL21 is produced by melanoma, and promotes immune tolerance	[227]
	CCL21 levels higher in patients with non-metastatic tumors	[228]
	HDAC inhibitors increase expression of CCR7	[229]
	CCR7 expression in primary melanoma and sentinel lymph nodes	[230]
	Footpad injection of B16 ± CCR7 had no effect on tumor growth, but reduced anti-tumor immunity	[231]
	VEGF-C induced CCL21/CCR7-mediated lymphangiogenesis which promoted the entry of naïve T cells into melanomas	[232]
	CCR7 overexpression shortened survival times/normal CCR7 increased overall survival	[233]
	CCR7 overexpression correlated with metal-binding protein	[234]
	CCR7 is typically cytoplasmic with <2% in the membrane	[235]
	PD-L1 and galectin-9 co-expression with CCR7 correlates with increased metastases	[236]
	Elevated expression of CCR7 in uveal melanoma did not affect LN metastasis. Observed elevated liver metastases correlated with CCR7	[237,238]

### 6.2. Skin Cancer

Although non-melanoma skin cancer occurs far more frequently than melanoma, most studies on the role of CCR7 in skin cancer have focused on melanoma. The limited information regarding non-melanoma skin cancer will be considered before discussing melanoma skin cancer. Immunohistochemical analysis of chemokine receptor expression patterns in non-melanoma skin cancer demonstrated downregulation of CCR6 and upregulation of CCR7 and CXCR4 in potentially metastatic non-melanoma skin cancer. This distribution did not exist in non-melanoma skin cancer with no metastatic potential, basal cell carcinoma, or actinic keratosis, when compared with normal skin, suggesting a possible role for CCR7 in non-melanoma skin cancer metastasis [222].

The well-established B16 murine melanoma model is frequently used as a proxy for human melanoma. B16 transduced with a CCR7-expression retroviral construct was injected into the footpad of mice and compared to control constructs. Within one week, elevated levels of tyrosinase-related protein-1 mRNA, a marker of melanocytes, were found in the draining lymph nodes for CCR7-expressing mice compared to controls. By three weeks post injection, melanoma metastasis was evident in >50% of mice expressing CCR7 relative to 5% in controls. A CCL21 neutralizing antibody prevented lymph node metastasis of CCR7-B16 cells unlike a control immunoglobulin [223]. These results suggested that CCR7/CCL21 mediated lymph node spread of B16 melanoma. Another study injected retroviral CCR7 and control vectors into the ear skin and footpads of mice and reported that there was no significant difference between CCR7+ tumors and controls during early tumor development, but control tumors receded by day 11 whereas the CCR7+ tumors formed visible nodules, which was accompanied by the presence of melanocytes in cervical lymph node by 21 days post-injection, suggesting that CCR7 enabled enhanced tumorigenesis and lymphoid metastasis [224]. Overexpression of CCR7 in non-metastatic melanoma cell lines led to these cells migrating towards lymphatic endothelial cells. In vivo studies using B16 melanoma cells injected into nude mice showed that CCR7-expressing melanoma cells migrated more efficiently to lymphatic tissue in response to CCL21 that could be inhibited by a CCL21 neutralizing antibody [225].

In vitro experiments showed that A375 cells derived from a malignant melanoma expressed CCR7, producing chemotaxis towards CCL21, which was prevented by CCL21 neutralizing antibodies, such as B16 melanoma cells [223,225,226]. When A375 cells were xenotransplanted into nude mice, the melanoma cells migrated to nearby lymphatic endothelial cells, which did not occur for non-metastatic melanoma cells. In this case, CCR7-expressing malignant melanoma cells were undergoing chemotaxis to CCL21 produced by lymphatic endothelial cells [31]. In a follow-up study, Shields et al. showed that melanoma tumors in mice produced CCL21 and initiated an immunotolerant milieu; in contrast, CCL21-deficient tumors induced antigen-specific immunity, suggesting that CCL21-related immune responses were facilitating cancer progression [227].

Analysis of several melanoma cell lines and tumor samples showed heterogeneous CCR7 expression as measured by quantitative real-time reverse transcriptase PCR, with CCR7 levels corresponding to CCL21 migration [228]. Curiously, analysis of patient sentinel lymph nodes from melanoma patients showed that CCL21 mRNA levels were higher in non-metastatic samples compared to lower CCL21 expression in patients with metastatic tumors, suggesting that CCL21 levels are downregulated during metastatic progression, possibly to reduce recruitment of CCR7-responsive immune cells [228]. Additionally, using melanoma cell lines, the potential role of epigenetic modifications was assessed by treating cells with a histone deacetylase (HDAC) inhibitor, trichostatin A, or a demethylating agent, 5-aza-2-deoxycytidine. Both treatments, singly or combined, increased expression of CCR7 and CXCR4 in melanoma cell lines, which, despite resulting in inhibition of cell growth, led to enhanced migration towards ligands. Such effects suggest that epigenetic manipulations might inadvertently increase the metastatic capabilities of CCR7-expressing melanomas [229].

In human melanoma, VEGF-C expression in primary melanoma specimens had a significant correlation with sentinel lymph node-positive metastasis, particularly for thin melanomas. Surprisingly, there was no link between CCR7 expression in primary melanoma and sentinel lymph node positivity [230]. In a murine model of melanoma, when equal numbers of CCR7-overexpressing B16 mouse melanoma cells or control B16 cells were injected into footpads and tumor growth and protein expression were analyzed, tumor growth was not significantly different between CCR7+ melanoma and controls. However, in contrast to the aforementioned study, in this murine study, CCR7 overexpression was associated with lymph node metastasis [231]. The primary genes reduced by CCR7 activation were in the interferon-γ pathway, suggesting downregulation of host anti-tumor immunity. In addition, increased VEGF-C and CCL21 staining was found in CCR7-expressing melanoma relative to control melanoma, further demonstrating a role for VEGF-C in lymphangiogenesis in mice [231]. More recently, using the B16 melanoma model, VEGF-C-induced CCL21/CCR7-mediated lymphangiogenesis that promoted the entry of naïve T cells into melanomas and thereby, enhanced the activity of immunotherapy [232]. 

In 38 patients with cutaneous melanoma, CCR7 overexpression was significantly linked to shorter time to progression and survival times, although, interestingly, normal CCR7 levels were associated with elevated overall survival [233]. In addition, CCR7 overexpression was associated with upregulated levels of the metal-binding protein, metallothionein, which had previously been identified as a risk factor for melanoma [234]; however, there was no correlation between CCR7 expression and lymph node metastasis in this study [233]. As reported with other types of cancer, CCR7 is often expressed in melanoma cell lines, whether from the primary tumor or metastatic lesions; however, CCR7 is typically found in the cytoplasm with little to no CCR7 found in the cell membrane (<2%), again questioning the functionality of the chemokine receptor [235]. A more recent study looked at CCR7 expression in 10 human melanoma cell lines derived from different anatomical locations. Between 1 and 5% of melanoma cells per cell line was identified as highly CCR7 positive co-expressed with two immune checkpoint ligands, PD-L1 and galectin-9, and had cancer stem cell characteristics. These cells were recognized and removed by natural killer cells but their accumulation was associated with a more metastatic phenotype driven by CCR7 [236].

Uveal melanoma is the most frequently occurring primary intraocular cancer, with an incidence of 6–7 cases per 1 million inhabitants per year in Western countries [239], and approximately half of these patients with uveal melanoma will have metastasis, with the liver being the most common site [240]. Analysis of primary uveal melanoma cell lines found elevated expression of CCR7 and CXCR4, although metastatic cell lines had no change in these receptors, and similar results were observed in a nude mouse model [237]. Further analysis suggested that liver-derived factors induced downregulation of CCR7 and CXCR4, although it was thought that, at least for CXCR4, downregulation likely occurred after metastasis since liver is a prominent source of CXCL12 [237]. Another study on uveal melanoma on paraffin-embedded tissue samples confirmed expression of CCR7 and CXCR4 and found expression of CCR10, primarily in the cytoplasm, for each chemokine receptor; CCL19 demonstrated a moderate expression [238]. This study found a significant correlation between CCR7 upregulation and liver metastasis, although the mechanism is not clear, particularly since CCR7 receptor was present at low levels on the cell membrane [238]. Further work looked at primary uveal melanoma specimens from metastatic patients who developed liver metastasis (19) and non-metastatic (30) patients, and data was also correlated to overall survival. There was strong cytoplasmic staining for CCR7 in 76% of patients with liver metastasis and 0% for non-metastatic patients with no evidence of any gene mutations. CCR7 expression also correlated with poor overall and disease-free survival [241]. Overall, data linked CCR7 expression to liver metastasis of uveal melanoma, but, as with the earlier studies, it is not clear that CCR7 is functional.

CCR7 is more commonly associated with melanoma rather than non-melanoma skin cancers. CCL21 and CCR7 were both produced by melanoma tissue promoting an immunotolerant phenotype. A role for VEGF-C in inducing CCL21/CCR7-mediated lymphangiogenesis of melanoma cells was noted and a low percentage of melanoma cells expressed the immune checkpoint ligands, PD-L1 and galectin-9, resulting in cancer stem cell characteristics and increased metastasis. CCR7 overexpression led to poor prognosis and reduced overall survival of patients although normal CCR7 ironically increased overall survival (Table 5).

## 7. Bone and Blood Cancers

### 7.1. Bone Cancer

Primary bone cancers are rare, making up less than 1% of all cancers, while metastasis of other cancers to the bone occurs frequently [35]. Overall, CCR7 expression is typically low in this type of cancer. Analysis of chemokine receptor expression in osteosarcoma cells found that 43% of the patient tumor samples expressed CCR7, although these levels were highly variable, such that CCR7 positivity per se did not correlate with overall patient survival; however, for high CCR7 expressers, there were statistically reduced overall survival and metastasis-free survival compared to low CCR7 expressors [242]. In a second study which examined the cells within the osteosarcoma tumor, although CCR7 expression was rare in osteosarcoma cells themselves, it was found in infiltrating inflammatory cells, which might account for the low lymphatic metastasis rates of osteosarcomas [243]. A similar result was found for the predominantly adolescent soft tissue/bone cancer, Ewing sarcoma, which is an aggressive cancer with a poor prognosis. Only one of twenty-four Ewing sarcoma patient tissues was positive for CCR7 or CCL21 [244].

Although CCR7 does not appear to be a significant factor in osteosarcoma or Ewing sarcoma progression, >80% of human chondrosarcoma (cartilage-initiated cancer) tissue samples had elevated CCR7 levels in conjunction with the EMT marker, transcription factor, Slug, resulting in a higher tumor grade and lower 5 year survival of patients [245]. In vitro experiments using SW1353 chondrosarcoma cells showed that CCL21-activated CCR7 induced an EMT phenotype with expression of phospho-ERK, phospho-AKT, Slug and N-cadherin [245]. Given the role that CCR7 has in other tumors, such as breast cancer, in promoting proliferation, it could be useful to examine the effect of knocking down CCR7 on the survival of bone tumors, although, as indicated previously, CCR7 does not appear to have a significant role in bone cancers (Table 6).

### 7.2. Leukemia

Adult T-cell lymphoblastic leukemia/lymphoma is a peripheral T-cell cancer, often fast-proliferating, manifesting frequently in the blood (leukemia) or as a lymphoma of lymph nodes, skin or other body sites and is primarily reported in Japan. The disease is highly linked to infections by human T-cell lymphotrophic virus type 1 (HTLV-1), although only a small percentage of infected individuals go on to develop cancer. Initial investigations in peripheral blood mononuclear cells from patients with adult T-cell leukemia revealed upregulated CCR7 mRNA expression. Additionally, T cells infected with HTLV-1 had increased CCR7 levels compared to uninfected T cells [246,247]. Adult T-cell leukemia patients with lymphoid involvement were more likely to express CCR7 than patients with no lymphoid association [248]. Next-generation sequencing was used to investigate single-nucleotide polymorphisms in adult T-cell leukemia/lymphoma patients from different regions of Japan, where it was found that similar mutation profiles occurred, including in the CCR7 gene, likely responsible for T-cell trafficking [249]. The results, however, contrasted with patient analysis from North America that showed a dominance of epigenetic modifications over genetic mutations [249]. Most of the CCR7 mutations in adult T-cell leukemia/lymphoma patients were truncations of the C-terminus cytoplasmic domain and were associated with elevated membrane localization of the CCR7 receptor, which the authors suggested was likely a gain-of-function mutation [250]. Adult T-cell leukemia cells were shown to be CD4^+^, CCR4^+^ and CD26^−^ and patients who had aggressive disease were also CCR7 positive, whereas indolent patients were negative for both CCR7 and CD127 (interleukin 7 receptor α) [251].

Acute lymphoblastic leukemia (ALL) affects both children and adults with B-ALL (B-ALL), constituting approximately 75% of cases, with the remainder being of the T-cell type (T-ALL). CCR7 was expressed in a high percentage of B cells from B-ALL patients and not expressed in control cord blood B cells [252]. B cells from B-ALL patients incubated with CCL19 prevented TNFα-induced apoptosis via CCR7-mediated stabilization of caspase-3 and caspase-8 and thus provided a pro-survival phenotype [252]. B-cell responses to CCR7 vary depending on the differentiation state of the cells. In childhood B-cell lymphoblastic leukemia cells, pre-B cells expressed CCR7 and migrated to CCL19, suggesting that CCR7 plays an important role in pre-B-cell lymphoblastic leukemia chemotaxis, whereas pro-B cells required the presence of the CD40 ligand to be CCL19/CCR7 responsive [253].

An unfortunate common complication of ALL is central nervous system (CNS) invasion often requiring intrathecal chemotherapy and cranial irradiation with associated significant morbidities. Notch-1-activating mutations occur in over 80% of T-ALL cases [254]. Using gene expression profile analysis, Buonamici et al. showed that CCR7 is downstream in the Notch-1 cascade [80]. In a mouse model, CCR7 was solely sufficient and necessary for T-ALL infiltration into the CNS, where the CCR7-expressing T-ALL cell line, CEM, was able to invade the brain, whereas the CCR7-negative cell line, DND41, did not invade the CNS. CEM-transplanted mice also had shorter survival times than for DND41-transplanted mice receiving the same number of cells. Furthermore, leukemic cells did not invade the CNS of plt mice in which CCL19 is inactive, possibly implicating the CCR7/CCL19 pathway in the CNS invasive phenotype, although these mice do have one of two CCL21 genes active [80]. The role of CCR7 in ALL CNS migration also appears to be relevant for the B-cell type in addition to the T-cell type. Zeta-chain-associated protein kinase 70 (ZAP70) is a 70 kDa CD3-receptor tyrosine kinase expressed in T cells and, to a lesser extent, in B cells [255]. A study investigated a potential role for ZAP70 in both B-cell and T-ALL invasion of the CNS. In 130 B-cell precursor ALL and 117 T-ALL patients, a correlation between ZAP70, elevated CCR7/CXCR4 levels and CNS invasion was observed [256]. The same study showed that ZAP70 regulated CCR7/CXCR4 via ERK1/2 activation and that short hairpin RNA knockdown of ZAP70 lowered CCR7/CXCR4 and reduced CNS B- or T-cell infiltration [256]. In a xenograft mouse model, CCR7 inhibition by itself was sufficient to drastically reduce CNS infiltration by ALL cells, indicating a key role for CCR7 in CNS manifestations of the disease [256]. 

The role of the Notch/CCR7 signaling pathway in T-ALL has been further investigated. Notch receptor activation signaling includes the PI3K/mTOR pathway, which has distinct complexes, mTORC1 and mTORC2. mTORC2 depletion attenuated CCR7 expression in T-cell leukemic cells, resulting in reduced tissue invasion and increased survival in Notch 1-linked T-ALL [257]. Mechanistically, mTORC2 acted through CCR7 and an Akt-dependent NF-κB pathway to modulate leukemia cell migration and survival [257].

In addition to acute leukemias, chronic leukemia progression has also been linked to CCR7 expression. Like acute leukemias, chronic lymphocytic leukemia can involve B cells or T cells, although B-cell chronic lymphocytic leukemia predominates, afflicting more than 95% of chronic lymphocytic leukemia patients, with approximately 1% of patients having T-cell prolymphocytic leukemia and a similar percentage diagnosed with B-cell prolymphocytic leukemia. This section will focus on the more prevalent B-cell chronic lymphocytic leukemia, but it is noteworthy that for T-cell prolymphocytic leukemia, which is characterized by rapid growth of mature post-thymic prolymphocytes and numerous cytogenetic alterations [258,259], CCR7 was highly expressed in T-cell prolymphocytic leukemia and associated with cell migration, survival and invasion. Blocking CCR7 with a monoclonal antibody abrogated these tumor characteristics and led to leukemia cell death in vitro and in a xenograft mouse model [260].

B-cell chronic lymphocytic leukemia (B-CLL), the most frequent adult low-grade lymphoproliferative disorder, is characterized by the accumulation of tumor CD5 B cells with enhanced survival potential. The peripheral blood B lymphocytes typically reach high numbers and these leukemic cells have an elevated propensity to invade lymph nodes, spleen and bone marrow [261]. It was noted that CCR7, CXCR4 and CXCR5 were highly expressed by malignant B-CLL cells and associated with cell migration to lymphoid tissues [262,263]. As mentioned, B-CLL cells have increased survival signaling. Similar responses were observed for B-CLL as described above for B-ALL, namely increased CCR7 expression that led to a block of TNFα-induced apoptosis and stabilization of caspases-3 and -8 and pro-survival responses when activated by CCL19 [252]. Inhibition of PI3K/Rho guanosine triphosphatase cascades led to a concomitant downregulation of CCR7 pathways in B-CLL cells, reducing cell migration and survival. Conversely, constitutively activated PI3K or RhoA elevated CCR7 B-CLL cells responses to CCR7 in the presence of CCL19 or CCL21. Interestingly, another potential downstream signal of PI3K, MAPK, was not involved in CCR7/CCL19/CCL21 migration/survival responses [264]. Another study identified the membrane remodeling F-BAR adapter protein, Cdc42-interacting protein 4 (CIP4), as a chemotactic regulator in B-CLL. CIP4 was highly expressed in B-CLL cells and, when stimulated by CCL19/CCR7, CIP4 binds to GTP-associated Cdc42 and interacts with the leading edge of the cell lamellipodia, stimulating the forward migration in JVM3 B-CLL cells in vitro [265]. The results suggested that CCL19 gradients produce dynamic changes in actin micro spike-containing lamellipodia facilitated by CIP4-modulated cell chemotaxis [265]. Further evidence of the importance of CCR7 in the migration and survival of B-CLL cells was shown in mice, where a murine anti-human CCR7 monoclonal antibody not only blocked cell migration to CCL19, but also promoted a complement-dependent cell cytotoxicity [261]. 

Although not discussed in detail here, CLL cells require proliferative and survival signals from lymphoid organs, including bone marrow. A particular subtype of B-CLL is characterized by trisomy 12, the most frequent chromosomal aberration in patients with B-cell CLL. This type of leukemia is noted for elevated levels of CD49d, the α4-subunit of the integrin VLA-4, a key regulator of leukemia cell homing to bone marrow and greater migration to the lymph nodes than other B-CLL [266]. This unexpected lymphoid tissue preference is likely due to high CD49d levels linked to decreased expression of the key B-cell migratory chemokine receptor, CXCR4. Furthermore, VLA-4 expressed in trisomy 12 B-CLL was more sensitive to CCR7, which likely explains the preference for lymph node migration rather than to bone marrow [267].

An important process in the progression of B-CLL is the infiltration of lymphoid tissues for which CCR7 and ligands are key chemokine receptors. As described previously for bladder and colorectal cancers, MMP-9, is produced by B-CLL cells, with overexpression frequently observed when the B cells infiltrate tissues [268]. CCL21/CCR7 activity increased MMP-9 production in B-CLL cells, which was inhibited by blockage of the ERK1/2 pathway, suggesting a role for the CCR7/MMP-9 pathway in B-CLL cell lymph node spread [269]. Another potential player affecting the CCR7 pathway and B-CLL cell migration is the atypical chemokine receptor, CCRL2 (ACKR5), for which CCL19 is a known ligand. CCRL2 is expressed on B cells in a maturation-dependent manner and is typically expressed by B-CLL cells [270]. Not surprisingly, B-CLL cells with high CCRL2 expression reduced CCR7/CCL19-induced B-cell migration, presumably since CCRL2 can bind CCL19, reducing its availability to interact with CCR7 [270]. Such interactions are likely part of the fine control mechanisms of B-CLL cell migration.

In addition to its function in ALL migration, ZAP70 also has a role in B-CLL chemotaxis, where it is highly expressed in aggressive forms of the disease [271,272]. ZAP70 was shown to increase B-cell receptor signaling and increase the responsiveness of ZAP70-positive B-CLL cells to CCL19 and CCL21 with enhanced migratory capacity toward CCR7 compared to ZAP70-negative cells, which was associated with F-actin polymerization, migration and lymph node involvement [271,272,273].

In addition to leukemia cell migration to lymphoid tissues, it is also important to consider the exit of these cells in controlling the overall exposure to pro-survival signals. The sphingosine-1-phosphate (S1P) receptor family are key regulators of T-cell and B-cell circulation [274,275]. S1P1 and S1P2 receptors were found to be responsible for B-cell egress from tissues into the blood and were downregulated in B-CLL [276]. The authors further showed that p66Shc, a Shc adaptor family member, increased S1P1 receptor expression in cells via a pro-oxidation mechanism; moreover, B-CLL cells were characterized by low reactive oxygen species, which correlated with the low S1P1 receptor levels. The authors also showed that p66Shc controlled expression of CCR7, but in the opposite direction to the S1P1 receptor [276]. Overall, the data supported a model in which p66Shc modulated by a low-oxidant milieu of B-CLL cells reduced S1P1 receptor levels, but enhanced CCR7 receptor levels such that the B cells were more likely to remain in lymphoid tissues in a pro-survival niche. In a later study, it was shown that the high cell surface levels of CCR7 and thus active localization observed in B-CLL cells are due to enhanced receptor recycling, which further accounts for the lymphoid tissue migratory capacity of these cells [277].

Other leukemias associated with CCR7 include chronic myelogenous leukemia (CML), which is characterized by the presence of the Philadelphia chromosome containing the BCR-ABL fusion gene [278]. Like several leukemias, CML is characterized by enhanced proliferation, cell survival and defective adhesion/migration and spleen enlargement is often observed [279]. The adhesion molecules L-selectin and ICAM1 are downregulated in CD34 progenitor CML cells, which at least in part explains the altered adhesive state. In addition, CCR7 mRNA levels were low with associated reduced responses to CCL19 and CCL21. The authors hypothesized that reduced CCR7 might explain the abnormal trafficking of CML cells [280]. The Ba/F3 murine interleukin-3-dependent pro-B cell line from a Balb/c mouse has been used to mimic CML cells. Signal transducing adaptor protein-2 (STAP-2) binds to BCR-ABL leading to enhanced BCR-ABL phosphorylation and downstream signaling. STAP-2 binding to BCR-ABL also enhanced CCR7, CCL19 and CCL21 expression via the MAPK/ERK pathway and was required for BaF3 cell growth in vitro [281]. When injected into nude Balb/c mice, small hairpin RNA inhibitor of CCR7 led to decreased tumor size compared to control Ba/F3, suggesting that CCR7 is required for the STAP-2/BCR-ABL-induced CML growth [281].

Overall, there is clear evidence that CCR7 plays important roles in leukemia migration. In T-ALL cells, CCL19 activation of CCR7 was sufficient to promote the entry of the leukemia cells into the CNS and for ALL patients, in general, ZAP70 was elevated along with CCR7 and CXCR4, which was also linked to CNS spread. In B-CLL, CCR7/CCL19 blocked TNFα-induced apoptosis to promote survival and CCR7 signaling through PI3K/Rho-promoted migration and survival. Furthermore, expression of the atypical chemokine receptor counteracts CCR7 effects by binding CCL19 and reducing B-cell migration. In adult T-cell lymphoblastic leukemia, infected T cells had increased CCR7 levels compared to uninfected T cells and were more likely to undergo chemotaxis to lymph nodes. This cancer uniquely possessed truncated CCR7, which appeared to be gain-of-function mutations (Table 6).

### 7.3. Lymphoma

Classic Hodgkin’s lymphoma (HL) is a lymphatic malignancy characterized by the presence of a minority of multinucleated Reed–Sternberg large cancerous lymphocytes, primarily derived from B cells that are surrounded by abundant inflammatory cells primarily consisting of infiltrating, T lymphocytes, histiocytes, eosinophils, and plasma cells [282]. It was speculated that chemokine receptors were likely key modulators of localization and all HL-derived cell lines tested expressed CCR7 and CXCR4 receptors with enhanced CCR7 expression mediated by NF-kB [283]. CCR7 expression varied depending upon the type of HL, with the classical subtype having mainly CCR7-positive cells located in the interfollicular zone of the lymph nodes, whereas the nodular lymphocyte-predominant subtype that associated with follicular structures had no CCR7 expression, although CXCR4 was highly expressed in this location [283]. 

Specimens from 41 patients with T-cell non-HL having lymphoid hyperplasia were investigated and showed elevated levels of CCR7 and MMP-9 correlating with increased numbers of cancerous lesions and higher tumor stage [284]. In vitro studies comparing Hut78 cells (cutaneous T-cell lymphoma) and Jurkat cells (adult T-cell lymphoma) revealed that CCR7 and MMP-9 levels were significantly higher in Hut78 cells compared to Jurkat cells, corresponding with elevated migration to CCL21 and suggesting that CCR7 plays a key role T-cell leukemia/lymphoma invasion [66,284].

Another type of B-cell-derived tumor is mantle cell lymphoma that usually originates from the clonal expansion of naïve CD5 B cells located in the mantle zone of secondary lymphoid follicles [285]. Mantle cell lymphoma cells express CCR7 and respond to CCL19 stimulation, whereas normal cells from the same tissue did not [286]. A subtype of large B-cell lymphoma, mediastinal large B-cell lymphoma, contains B cells sharing surface markers such as thymic-derived B cells, which respond to CCR7 signaling. Surprisingly, CCR7 expression in mediastinal large B-cell lymphoma cells was low to absent and overall chemokine profile was distinct compared to other lymphomas. In a later study, the same group used the Eμ-Myc transgenic mouse model in which the B-cell lymphoma traffics to the lymph nodes and spleen via activation of CCR7 [28]. CCR7 mediated chemotactic responses to the splenic T-cell zone that led to a significant survival benefit when compared to CCR7-deficient lymphoma cells. Furthermore, within the spleen niche, B-cell lymphoma cells interacted with gp38+ fibroblastic reticular cells, releasing homeostatic chemokines in response to CCR7 activation by CCL19 or CCL21 released by local stromal cells [28]. Epstein–Barr virus-positive diffuse large B-cell lympho-proliferative disorder not otherwise specified is a rare cancer, originally described in 2003 in elderly patients over the age of 60 [287]. Whole-genome sequencing identified recurrent mutations in several genes including CCR7 in 11% of cases, which was proposed to be a mechanism for homing of the lymphoma cells to secondary lymphoid tissues, where the Epstein–Barr virus can propagate and further expand the lymphoma growth [288,289].

A rare cancer that is most often associated with human immunodeficiency virus infection is primary central nervous system lymphoma, which is a form of non-Hodgkin’s lymphoma found only in the central nervous system, which is typically an unusual niche for B cells [290]. Using primary central nervous system lymphoma tissue, CCR7 was expressed in all 29 samples tested, along with CXCR4 and CXCR5; in each case, chemokine receptor staining was found only in the cytoplasm, with no membrane staining observed. In contrast, CCR7, CXCR4 and CXCR5 staining was both cytoplasmic and membranous for all 29 peripheral B-cell lymphomas [291]. A study showed that astrocyte-produced CCL19 was required for gliosis-induced central nervous system lymphoma progression. Deleting CCL19 in mice or CCR7 in lymphoma cells was sufficient to prevent central nervous system lymphoma development [292]. Two-photon microscopy in mice divulged that lymphoma cells transiently enter the brain parenchyma along a CXCL12 gradient; however, cell retention was enhanced by astrocyte-derived CCL19 stimulating central nervous system lymphoma genesis [292]. A similar mechanism is proposed in humans, where astrocytic CC19 was found in human gliosis and central nervous system lymphoma samples.

Multiple myeloma is a plasma B-cell malignancy of the bone marrow that is often heritable and associated with single-nucleotide polymorphisms [293]. Initial work determined that three gene variants were associated with multiple myeloma, which included CCR7; however, since CCR7 is not typically expressed in multiple myeloma and the specific single-nucleotide polymorphism was not linked to CCR7 gene expression, the authors cautioned if CCR7 genetic variants were involved in multiple myeloma [294].

Cutaneous T-cell lymphoma consists of a several variants including Sézary syndrome and the more common, mycosis fungoides. Expression of several chemokine receptors was elevated in cutaneous T-cell lymphoma, primarily Sézary syndrome samples, which included CCR7. CCR7 levels correlated with T-cell lymphocyte presence in the epidermis [295]. Mycosis fungoides is characterized by a clonal expansion of atypical CD4+ skin-homing T lymphocytes [296]. CCR7 was expressed in 62% of mycosis fungoides patient samples that correlated with the subcutaneous skin expansion of the lymphoma cells [297]. Elevated CCR7 presence was also found on the membrane of MyLa cells derived from a patient with mycosis fungoides that showed migration to CCL21 mediated via the mTOR pathway [297]. Typically, mycosis fungoides is a slow-growing tumor of the skin, although it can metastasize to lymph nodes and visceral organs in the advanced stages. Long non-coding RNA metastasis-associated lung adenocarcinoma transcript 1 (MALAT1) was especially increased in mycosis fungoides tissue. Using MyLa cells, CCL21 increased migration and enhanced mTOR activation, as previously reported. These effects were reversed by MALAT1 knockdown and indicated that MALAT1 is an important mediator of CCL21/CCR7 migratory actions in mycosis fungoides [298]. 

Similar to acute lymphoblastic leukemia, primary CNS lymphoma cells expressed CCR7, with CNS entry being dependent on CCR7 and its ligand, CCL19. Hodgkin’s lymphoma migrated to the interfollicular zone of lymph nodes and expressed CCR7, which was dependent on NF-kB. Cutaneous T-cell lymphoma expresses high levels of CCR7 compared to adult T-cell lymphoma, which are associated with retention of the lymphoma cells in the skin (Table 6).

**Table 6:** Hematopoietic/bone.

Cancer	Observation	Reference
Bone	CCR7 expression is low (expressed in 43% of patient samples); high CCR7 reduced survival	[242]
	Osteosarcoma—CCR7 expression is rare/low lymph metastases.	[243]
	Ewing sarcoma CCR7 or CCL21 expressed in ~4% of patient samples	[244]
	More than 80% of chondrosarcoma tumors co-expressed CCR7/Slug (EMT marker); reduced 5 year survival. SW1353 chondrosarcoma cells phosphorylated ERK, AKT and expressed Slug and N-cadherin following CCL21 stimulation	[245]
Adult T-cell lymphoblastic leukemia/lymphoma	HTLV-1-infected T cells had increased CCR7 levels compared to uninfected T cells, and were more likely to traffic to lymph nodes	[246,247,248]
	SNPs common in CCR7 or epigenetic modifications may promote T-cell chemotaxis	[249]
	CCR7 expression and GOF CCR7 mutations linked to more aggressive disease	[250,251].
Acute lymphoblastic leukemia (ALL)	CCR7 expressed in B cells from B-ALL patients, but not in control cord blood B cells	[252]
	B cells activated with CCL19 blocked TNF-α-induced apoptosis	[252]
	Childhood B-cell lymphoblastic leukemia cells, pre-B cells express CCR7 and migrate to CCL19. Pro-B cells required CD40 to be CCL19 responsive	[253]
	CCR7 expression in pediatric T-ALL necessary and sufficient for CNS invasion	[80]
	In 130 B-cell precursor ALL and 117 T-ALL patients, a correlation between ZAP70, elevated CCR7/CXCR4 levels and CNS invasion; Xenograft supported this observation	[256]
	In a mouse model, CCL19 and CCR7 promote CNS invasion of lymphoma	[292]
	mTORC2 promotes T-cell invasion, cell migration and survival	[257]
	CCR7, CXCR4 and CXCR5 were highly expressed in B-CLL	[262,263]
	B-CLL active via CCR7/CCL19 blocked TNFα-induced apoptosis to promote survival	[252]
	In B-CLL CCR7 signals through PI3K/Rho to promote migration and survival	[264]
	CCL19/CCR7 promotes Cdc42 activation during chemotaxis in JVM3 B-CLL cells	[265]
	trisomy 12 B-CLL demonstrates preferential migration to LN	[267]
	CCL21/CCR7 activity increased MMP-9 production in B-CLL	[269]
	CCRL2 competitively inhibits CCR7/CCL19-induced B-cell migration	[270]
	p66Shc controlled expression of CCR7, promoted retention in LN	[276,277]
	CML has low CCR7 levels that correlate with abnormal trafficking of CML	[280]
	CML signals via CCL19/CCL21 to promote cell growth	[281]
Lymphoma	HL expressed CCR7 via NF-kB and promoted migration to interfollicular zone of LN	[283]
	T-cell non-HL expressed high levels of CCR7 and MMP-9	[284]
	Cutaneous T-cell lymphoma (HuT78) expresses high levels of CCR7 compared to adult T-cell lymphoma (Jurkat)	[284]
	Mantle cell lymphoma express CCR7 and migrate to CCL19	[286]
	Mediastinal large B-cell lymphoma responds to CCR7 signaling	[299]
	CCR7 directed chemotaxis of Eµ-Myc B-cell lymphoma promotes survival	[28]
	CCR7 is mutated in ~11% of Epstein–Barr virus B-cell lymphoproliferative disorders.	[288,289]
	100% of primary CNS lymphoma cells expressed cytoplasmic CCR7, while CCR7 was membrane and cytoplasmic in B-CLL; deleting CCR7 or CCL19 prevented CNS lymphoma	[291,292]
	Cutaneous T-cell lymphoma (Sézary syndrome) cells expressed CCR7, correlates with presence in epidermis	[295,297]
	In MyLa cells, CCL21/CCR7 increased migration and enhanced mTOR activation	[298]

## 8. Discussion

CCR7 is a pivotal chemokine receptor for cell migration to secondary lymphoid organs, thus it is not surprising that tumor metastasis to lymph nodes was elevated when CCR7 expression was enhanced for all types of cancer. Tumors that typically show low metastasis to lymph nodes, such as prostate cancer, also had a propensity to migrate to lymph nodes in cases where CCR7 expression was identified [101,102,105]. Actin dynamics are important for cell migration and at least for head and neck cancers and chronic leukemias, CCR7-activated actin filament accumulation at the cell membrane by stimulation of small GTPases, RhoA and Cdc42, leading to lamellipodia, membrane ruffling and promotion of cell migration and invasive behavior [188,189,264,265]. Inhibition of CCR7 blocked another small GTPase, Rac, with concomitant decreased actin polymerization and reduced head and neck cancer migration, further indicating an important role for CCR7 in actin dynamics and cell migration [183]. By activating either the T-cell receptor for T-CLL or the B-cell receptor for B-CLL, CCR7 induced expression of the protein tyrosine kinase, ZAP70, increasing leukemia cell actin polymerization and migration, suggesting an alternative CCR7-linked actin-dependent migratory pathway in leukemia [256,271,272,273]. 

Tumor lymphangiogenesis is an important process to facilitate tumor metastasis to lymphatic vessels. Several studies indicated that CCR7-induced lymphatic migration is related to changes in the expression of key VEGF family lymphangiogenic factors. Increased VEGF-C and CCR7 expression in breast carcinoma tissue and increased CCL21/CCR7 activation in lymphatic endothelial cells promoted lymphatic endothelial cell proliferation and lymph node metastasis of the CCR7-expressing breast cancer cells [68]. In head and neck cancers, VEGF-C and CCR7 co-expression correlated with lymph node metastasis [160]. In CCR7-expressing melanoma cells, VEGF-C and CCL21 were expressed by lymphatic vessels and enhanced lymph node metastasis compared to non-CCR7-expressing control tumors [231]. VEGF-C and VEGF-D expression correlated with CCR7 levels and was associated with esophageal and lung cancer progression, respectively, with increased lymph node metastasis and reduced patient survival [126,212,300]. Furthermore, approximately half of gastric cancers co-expressed VEGF-C and CCR7 and sometimes VEGF-D that strongly predicted lymph node metastasis [137,138]. Despite the primarily poor patient survival when CCR7 or ligands and VEGF were co-expressed in cancers, at least for some colorectal patients, there was discord between a CCR7 ligand and VEGF, where increased CCL19 levels were positively associated with patient survival, which to some extent was linked to decreased VEGF-A expression [118]. Nevertheless, it is evident that CCR7 and VEGF are commonly co-expressed in a variety of different cancers, which are poor prognostic factors typically resulting in lymphatic invasion and reduced patient survival.

Specific cell invasion of the CNS via CCR7 activation was shown for some leukemias/lymphoma, including human T-ALL, B-ALL and primary central nervous system lymphoma [80,256,301]. The mechanisms of leukemia/lymphoma CNS cell invasion were CCR7 dependent and involved distinct pathways. Using a mouse xenograft model of T-ALL, Buonamici et al. showed that CCR7-induced CNS invasion was contingent on CCL19/CCR7 activation, which was downstream of Notch-1 and included activation of the PI3K and mTOR pathways [80]. For CCR7-dependent B-ALL, CNS entry was linked to ZAP70 activation, as described previously [256], whereas, for primary central nervous system lymphoma, astrocyte-derived CCL19 retained CCR7-expressing lymphoma cells in the CNS, leading to gliosis and increased risk of gliosis-induced primary central nervous system lymphoma [292]. These data strongly support the concept that CCR7 is an important mediator of leukemia cell entry into the CNS, although the exact mechanism(s) are unclear and might be multifaceted.

In addition to cell migratory effects, CCR7 was often associated with increased cancer cell survival including tumors of the breast, gastrointestinal, gynecological, head and neck, lung cancer and leukemia. The mechanisms of CCR7 enhanced cell survival were varied. CCR7 expression in triple-negative breast cancer cells led to reduced anoikis and increased tumor cell survival [70], with concomitant activation of ERK and Akt signaling [71]. In bladder cancer, CCR7 enhanced pro-survival Bcl-2, while decreasing pro-apoptotic Bax proteins [87]. In gastric cancer, tumor cell CCR7 expression resulted in an increased Treg population and elevated tumor cell survival [133]. In head and neck cancers, CCL19/CCR7 activation produced phosphorylation of mTOR that elevated cell survival [180,302]. In B-ALL, the presence of CCL19 reduced TNF-α-induced apoptosis by stabilizing caspase-3 and caspase-8 [252]. In T-ALL, mTORC2 was shown, like CCR7, to be downstream in the Notch-1 signaling pathway that activated the Akt-dependent NF-κB pathway to modulate leukemia cell survival [257]. It can thus be concluded that CCR7 activity often elicits pro-survival responses in cancer cells and poor patient prognosis.

CCR7-enhanced migratory ability is, at least in part, due to EMT characteristics that were observed in several cancers including breast, bladder, bone, colorectal, gastric, head and neck, lung, pancreatic, prostate, thyroid, B-CLL and non-Hodgkin’s lymphoma. Although different markers were used to evaluate EMT in cancers, there was a consistent CCR7-induced increased activation of Slug, N-cadherin, TGF-β, vimentin, phospho-ERK, phospho-Akt, MMP-2, MMP-9 and Snail attenuation of E-cadherin [75,76,87,104,119,142,148,199,209,245,269,284,303]. For solid tumors, CCR7 induced EMT enhanced tumor cell migration and invasive behavior. For B-CLL, CCR7-enhanced expression of MMP-9 was associated with increased migration of leukemia cells to lymph nodes and for non-Hodgkin’s lymphoma to an increase in cancerous lesions [199,284]. Often linked to an EMT phenotype in solid cancers is the presence of hypoxic conditions. In vitro studies showed that HIF-1, which is induced under low oxygen tension, increased the expression of CCR7 in breast cancer, ovarian epithelial and head and neck cancer cells [36,95,193]. In vivo studies showed that hypoxia-induced CCR7 expression was associated with a more invasive EMT-like phenotype [140,193].

TGF-β signaling is a key activator of EMT and can be stimulated by CCR7 activation. In gastric cancers, increased CCR7 led to increased TGF-β1 and EMT [141]. In breast cancer, TGF-β1 activates a Smad protein-linked pathway, resulting in EMT phenotype and lymphatic migration in response to CCL21 release from lymphatic endothelial cells and chemotaxis of CCR7-expressing breast cancer cells [303]. TGF-β1 activates the serine/threonine kinase, TGF-β-activated protein kinase 1 (TAK1), which is a regulator of proinflammatory and innate signaling pathways, leading to increased CCR7 and inflammation/increased cell survival [304]; indeed, it was shown that activation of TAK1 increased expression of CCR7 in triple-negative breast cancers and enhanced EMT, lymph node invasion and cancer cell survival [72]. Conversely, in lung cancer, decreased CCR7 led to decreased NF-κB and decreased TGF-β1, which reduced EMT [207]. Another common inflammatory mediator, COX-2, is likely upstream in a pathway that affects CCR7 expression. Elevated COX-2 in breast cancer cells led to increased CCR7 and enhanced migration of breast cancer cells to lymphatic endothelial cells and lymph node metastasis [47,48]. The results suggest a link between inflammatory mediators, CCR7, EMT and cancer progression. 

The cytoplasmic adapter protein, CrkL, is integrated into tyrosine kinase signal cascade pathways in humans in response to diverse stimuli that can lead to cancer susceptibility [305]. CCL19/CCR7 activation increased CrkL levels in ovarian epithelial carcinoma, which correlated with EMT and lymph node metastasis; conversely, CrkL knockdown decreased CCL19/CCR7 activation and reduced EMT [100]. At least for gynecological cancer, CrkL was shown to be a regulator of alternative splicing of several genes involved in cancer progression, although it was not clear that CCR7 underwent alternative splicing that could be related to cancer progression [306] Results for CrkL are not consistent since, in lung cancer, high CCR7 mRNA was co-expressed with CrkL and c-ABL expression, which in this case led to better prognosis [219]. Additionally, elevated CCL19 was a good prognostic factor in lung adenocarcinoma patients, suggesting that CCR7/CrkL interactions are variable and might be tumor type specific [219].

CCR7 gene mutations have been linked to cancer progression. Two SNPs increased risk of NSCLC [218]. In colorectal cancer, truncated CCR7 led to growth and survival advantages of cancer cells [115]. In adult T-cell leukemia/lymphoma, evidence suggests that both genetic mutations and epigenetic changes are involved in cancer progression. Common CCR7 SNPs were found in Japanese adult T-cell leukemia/lymphoma patients, although CCR7 differences were typically epigenetic changes rather than gene mutations in North American patients [249]. In addition to SNPs, most of the larger CCR7 mutations in adult T-cell leukemia/lymphoma patients were truncations of the C-terminus cytoplasmic domain, which were likely gain-of-function mutations [250]. In melanoma cells, inhibitors of the epigenetic modifiers histone deacetylation or demethylation led to increased CCR7 expression and to increased cell migration [229]. Again, results are not necessarily one sided since CCR7 gene mutations can be beneficial in cancer since, in breast cancer samples, CCR7 splice variants increased patient survival [85].

MiRNAs function in the regulation of gene expression and can be dysregulated in cancer. MiRNAs may function as either oncogenes or tumor suppressors under certain conditions [307]. Studies identified miR-let-7a and miR-335 as likely regulators of CCR7 based on targeted interactions identified by the MirTarBased database [216]. In breast cancer, miR-let-7a did indeed reduce CCR7 levels with concomitant attenuation of breast cancer cell migration and invasion [83]. Similarly, in gastric cancers, a reduction in miR-let-7a activity led to increased CCR7 expression, gastric cancer progression and metastasis [139]. In lung cancer, miR-335 and miR-let7a had opposite effects. Lung cancer patients with lymphoid metastatic involvement had higher expression of miR-335 and lower expression of miR-let7a associated with increased CCR7 expression and increased cell migration [216]. In head and neck cancers, another miRNA member of the let 7 family, hsa-let-7e-5p reduced CCR7 levels; conversely, inhibition of hsa-let-7e-5p increased CCR7 expression and elevated cell proliferation, migration and invasion [197]. These results suggest that let seven family miRNAs act as tumor suppressors, at least in part, by inhibiting CCR7 activity. Another miRNA, hsa-miR-125a-5p, upregulated CCR7 expression, cell proliferation, migration and invasion in a head and neck squamous carcinoma cell line, in line with observed patient survival results [194,195]. Similarly, another microRNA, miR-1275, also elevated CCR7, leading to a more aggressive cancer cell phenotype [196]; and, in bladder cancer, decreased miR-199a-5p led to increased CCR7 and increased MMP-9 and elevated cell migration [88]. Overall, data supports roles for miRNA playing an important role in controlling CCR7 expression and cancer progression.

CCR7 is expressed on several cell types, in particular cells of the immune system. CCR7 ligands, CCL19 and CCL21 are primarily expressed in secondary lymphoid organs and play pivotal roles in the chemotactic migration of CCR7-expressing immune cells to the secondary lymphoid organs that are important components of adaptive immune responses. Cancer cells that express CCR7 behave in a similar fashion to immune cells, such that they also follow chemotactic gradients that lead to lymph node migration and metastasis. In one case, immune response, CCR7 activity is typically beneficial, whereas in another other case, cancer spread, CCR7 responses are detrimental to the individual. This poses a quandary when considering antagonizing CCR7 as potential anti-cancer therapy, although specific targeting of CCR7 may be possible that does not adversely affect immune responses. Indeed, pioneering pre-clinical studies using CCR7 function-blocking single-chain antibodies demonstrated that these pre-clinical biologicals blocked T-ALL cell transmigration across a human brain endothelial cell monolayer, and Ca^2+^ mobilization in vitro [308]. More recently, an anti-CCR7 antibody was used to reduce CLL migration to the lymph nodes and the anticipated clinical trials will further examine the efficacy/safety of this anti-CCR7 therapy [309]. Moreover, systemic anti-CCR7 therapy might be warranted if the benefits outweigh the risks of compromising normal immune responses, which will depend on the length of therapy to some extent. In fact, such an approach might be favorable since murine studies have determined that the absence of CCR7 ligands results in delayed but enhanced T-cell responses [156]. Time will tell if CCR7-based therapeutic approaches provide significant cancer patient benefits.

## Figures and Tables

**Table 1:** Breast cancer and CCR7.

Signaling Event	Observation	Reference
CCR7/CCL21	Promotes migration/invasion via activation of actin	[32]
CCR7/EGFR	Shortened patient survival time, high local recurrence; no difference in 5 year survival.	[60,61]
CCR7/HER2-neu	Correlates with LN metastases	[65]
CCR7 in inflammatory BCA	Decreased 5 year survival	[55]
CCR7 Mutations in basal-like breast cancer	Site-dependent reduction or promotion of disease progression or survival	[85]
CCR7 targeting	Promoted bone or skin metastases	[56,57]
Cell death	Anoikis inhibitor, TAK1 promotes CCR7 expression/tumor growth	[70,71,72]
EMT	TGF-β1 induced EMT	[75,76,77]
Endothelin (ET-1 activation)	May upregulate CCR7 expression via HIF-1; may promote invasion	[36]
Ets-1	Promotes CCR7 expression in TBNC	[50]
CCR7 expressed by spindle-shaped stromal cells	Not associated with patient survival	[59]
Luminal A Breast cancer	Does not promote CCL21 chemotaxis	[51]
Luminal B and TNBC breast cancer	High levels of CCR7 promotes Notch-linked tumor formation	[33,52,53,78]
miR-let-7a	In patient cells and cell lines prevents chemotaxis and invasion	[83]
Prostaglandin E2 (PGE2)/EP2 and EP4	PGE2 via COX-2 leads to AKT-mediated phosphorylation of SP1; SP1 binds CCR7 promoter to increase levels.	[44,48]

**Table 2:** The roles of CCR7 in genitourinary cancers.

Cancer	Observation	Reference
Bladder	CCR7 not associated with aggressive form	[86]
	miR-199a-5p represses CCR7 expression in normal tissues and it is reduced in bladder cancer; reduction correlates with increased MMP-9	[88]
	RRBP1 knockdown increased CCR7	[89]
Gynecologic	Cervical squamous cell	[93]
	Cervical squamous cell—elevated CCL19 blocked apoptosis	[94]
	Ovarian cancer—CCR7 reduced survival (cytoplasmic or nuclear)	[93]
	Ovarian cancer epithelial cells, SKOV-3, revealed elevated CCR7 in hypoxia; CCL21 promoted EMT/invasion	[95]
	CrkL induced by CCL19/CCR7 correlates with higher-stage, lymph node metastasis and reduced overall survival; CrkL knockdown attenuates EMT in SKOV-3 cells	[100]
	Differential gene analysis of squamous cell carcinoma demonstrated CCR7 in combination with PD-1, ZAP-70 and CD28 led to improved immune-mediated 5 year overall survival	[97,98,99]
Prostate	Strong lymph node staining for CCR7 may correlate with LN metastases	[101,102]
	CCR7 siRNA in PC-3 cells inhibits VEGF and MMP along with tumor size in xenograft	[103]
	Exogenous expression of CCR7 in PC-3 increased Notch1, pMAPK, pp65, MMP-9, N-cadherin and Snail (EMT) to enhance migration/invasion	[104]
	Pro-inflammatory cytokine TNF-α induced CCR7 in cell lines, which induced p38 MAPK phosphorylation.	[105]

**Table 3:** CCR7 in Gastrointestinal cancers.

Cancer	Observation	Reference
Colorectal Cancer (CC)	CCR7 expression elevated in ulcerative colitis (UC). 5 year survival reduced by CCR7. siRNA knockdown of CCR7 in SW620 human CC cells injected into athymic nude Balb/c mice reduced invasion and LN metastases.	[109]
	Variable CCR7 expression in colorectal cancer.	[112]
	CCR7 elevated in tumors of the rectum.	[112]
	Truncated/nonfunctional mutants of CCR7.	[113]
	Truncated CCR7/reduced CCL21.	[115,116]
	Increased CCL19 correlated with increased survival.	[117]
	CCR7 correlates with increased MMP-9 in human SW480 cells. shRNA knockdown of CCR7 reduced metastasis and increased survival.	[119]
	CCR7 upregulated by COX-2.	[120]
	CCR7 had no effect on patient survival; however, co-expression of CCL21 receptor, CXCR3 correlated with metastases.	[122]
	Co-expression of CCR7 with EGFR generated cetuximab-resistant EGFR; this was reduced by expression of CCL21.	[123]
Esophageal	Expressed in 45% of esophageal squamous cell carcinoma; activated by CCL21. Expression correlates with decreased survival.	[124]
	LN metastasis, but not primary tumor expressed high levels of CCR7.	[125]
	Co-expression of CCR7/VEGF-C mRNAs worsened prognosis/survival compared to non-expressors.	[126]
	Co-expression of CCR7/MUC1 correlates with poor prognosis. MUC1 inhibition blocks CCL21-induced invasion.	[127]
	Murine model CCL21/CCR7 led to metastases.	[128]
Gastric	~84% or patient samples express CCR7 (RT-PCR) but only 23% stain CCR7 positive with anti-CCR7.	[130]
	~65% of patient samples expressed CCR7 which correlated with LN metastases (RT-PCR and IHC). In vitro gastric carcinoma cell lines (66%) migrated to CCL21.	[131]
	~30% of patient samples expressed CCR7.	[132]
	~70% of patient samples expressed CCR7, which correlated with LN metastases.	[133]
	*H. pylori* upregulated CCR7 in gastric epithelium.	[135]
	Transformation of *H. pylori*-linked gastritis to MALT lymphoma or B-cell lymphoma which expressed CCR7.	[136]
	VEGF-C/CCR7 co-expression in ~50% of cancers.	[137]
	VEGF-C, VEGF-D or CCR7 predicted lymphatic invasion of gastric tumor.	[138]
	HIF-1α upregulates CCR7/increases COX-2 and MMPs.	[140]
	Gastric cancer mediated by CCR7 by inducing EMT via TGF-β1.	[141]
	CCR7 correlates with Snail, which represses E-cadherin to promote EMT.	[142]
	Meta-analysis indicates that CCR7 significantly increases risk of lower 5 year overall survival rate for CCR7+ vs. CCR7- gastric cancers (HR = 0.46, 95% CI 0.31–0.70. *p* < 0.001).	[143]
Pancreatic	CCR7 regulates Twist to promote EMT.	[148]
	CCR7 levels are high, and ligand CCL21 is low in pancreatic cancer tissues compared to normal pancreas.	[145]
	CCR7 induced Twist in 72% of patient samples. May mediate EMT, tumor progression and LN metastasis.	[148]
	CCR7/CCL19 in PANC1 cells induced pERK, pAKT, N-caherin and MMP-9 (EMT).	[148]
	CCR7 elevated in CD133+ pancreatic cells via the ERK/NF-κB pathway.	[149]
	PANC1 cells induced MMP-9, ATM, and BRCA1 but downregulated CASP8 when stimulated with CCL21.	[150]
	CCL21 downregulates AKT1, FOS and JUN.	[150]
	CCL21 promoted sensitivity to pain.	[151]

**Table 4:** CCR7 in Head and Neck and Endocrine cancers.

Cancer	Observation	Reference
Oral	~65% were positive for CCR7/correlated with tumor progression, large lymph node metastases and reduced survival.	[154,155]
	B7E3 oral squamous cell carcinoma in mice had higher growth rate + CCL19/CCL21-ser than in *plt* mice lacking these ligands.	[157]
	No effect of CCR7, CCL19 and CCL21 mRNAs which are expressed in squamous cell carcinoma and normal oral mucosa.	[158]
	CCR7 activation in tongue squamous cell carcinoma cell line SCC4 led to more aggressive cancer, while inhibition reduced migration/invasion. No effect on cell growth.	[159]
	CCR7,VEGF-C and VEGFR-3 correlated with lymph node metastases in tongue cancer.	[160]
	CCR7 expressed at higher levels in male tongue cancer patients than females/no effect on prognosis.	[160]
	CCR7 is a biomarker of poor prognosis and shorter disease-free survival in tongue cancer.	[161]
	UCA long non-coding RNA is co-upregulated in conjunction with CCR7 in tongue squamous carcinoma cells—Correlates with increased proliferation, migration and glycolysis.	[162,163]
	Upregulation of CCR7 in squamous carcinoma of the tonsils led to poor prognosis.	[164]
Non-oral	High levels of CCR7 mRNA correlate with poorly differentiated tumors and lymph node metastases.	[167]
	CCR7 promotes progression.	[168,169]
	Twist expression leading to EMT is linked to CCR7 expression.	[170]
	Epithelial nasopharyngeal patient samples had heterogenous expression of CCR7 (CXCR4,CXCR6) in liver metastases.	[172]
	Nasopharyngeal cancer-high levels of CCR7 correlate with poor prognosis.	[173]
	CCR7 does not appear to contribute to lung metastases.	[174]
	CCR7 significantly increases migration in PCI-37B cell line (HNCC) and lymph node metastasis via integrin αvβ3 (*p* < 0.05). Effect mediated by Src.	[166,175,177]
	β-defensins induce CCR7 expression via NF-kB promote cell survival and migration.	[184,185]
	Autologous CCR7 stimulation of HNCC by CCL19 induced mTOR activation, via PI3K and JAK2/STAT3-mediated migration via activation of Cdc42.	[180,181,183]
	Autologous CCR7 stimulation of HNCC activates ERK1/2 and JNK phosphorylation and EMT.	[182]
Thyroid	CCR7 expression is 9-fold higher in papillary thyroid cancer and medullary thyroid cancer compared to follicular and poorly differentiated tumors.	[199]
	CCL21 activation of CCR7 in TPC-1 induced proliferation, migration, MMP-2, MMP-9 and increased β1-integrin expression.	[199]
	IHC staining of 65 patients—angiolymphatic invasive or tumors with LN metastasis had elevated CCR7 when compared to non-invasive tumors.	[200]
	Only 5–10% of cells express CCR7 in 30 patient samples.	[201]

## Data Availability

Not applicable.
